# Synthesis and properties of novel star-shaped oligofluorene conjugated systems with BODIPY cores

**DOI:** 10.3762/bjoc.10.285

**Published:** 2014-11-19

**Authors:** Clara Orofino-Pena, Diego Cortizo-Lacalle, Joseph Cameron, Muhammad T Sajjad, Pavlos P Manousiadis, Neil J Findlay, Alexander L Kanibolotsky, Dimali Amarasinghe, Peter J Skabara, Tell Tuttle, Graham A Turnbull, Ifor D W Samuel

**Affiliations:** 1 WestCHEM, Department of Pure and Applied Chemistry, University of Strathclyde, Glasgow, G1 1XL, UK; 2 Organic Semiconductor Centre, SUPA, School of Physics & Astronomy, University of St. Andrews, St. Andrews, KY16 9SS, UK; 3 Institute of Physical-Organic Chemistry and Coal Chemistry, 83114 Donetsk, Ukraine

**Keywords:** absorption spectroscopy, BODIPY, cyclic voltammetry, photoluminescence, star-shaped oligofluorene

## Abstract

Star-shaped conjugated systems with varying oligofluorene arm length and substitution patterns of the central BODIPY core have been synthesised, leading to two families of compounds, **T-B1–T-B4** and **Y-B1–Y-B4**, with T- and Y-shaped motifs, respectively. Thermal stability, cyclic voltammetry, absorption and photoluminescence spectroscopy of each member of these two families were studied in order to determine their suitability as emissive materials in photonic applications.

## Introduction

The boron-dipyrromethene (BODIPY) or 4,4-difluoro-4-bora-3a,4a-diaza-*s*-indacene unit has attracted much attention due to the properties it imparts to the compounds that contain it, such as efficient luminescence, high absorptivity, good photostability and solubility in common solvents [1–4]. Due to these properties, BODIPY-containing conjugated systems have found numerous applications as laser dyes [5–7], labels for biological imaging [8–10] and low band-gap polymers [11–13]. Being efficient emitters in solution, small molecule BODIPY compounds often show signs of aggregation at high concentration and in solid films [14–15], which lowers the luminescence efficiency of the material. Due to this aggregation and a low Stokes shift, BODIPY dyes with efficient emission in the solid state are still rare [16].

One of the strategies to suppress the aggregation of π-functional emissive units is to incorporate them into star-shaped structures [17]. A multidimensional molecular architecture provides excellent film-forming properties of the materials and an isotropic morphology of the final film. In the case of oligofluorenes with benzene [18] and truxene cores [19–22], this design methodology has yielded promising materials for lasing applications. Previously, we have designed star-shaped systems featuring energy transfer by incorporating acceptor chromophore units either within the arms [23], or in the core [24], of parent blue-emissive star-shaped oligofluorene systems. This strategy provides the ability to tune emission and create materials with an increased separation between absorption and emission profiles, which makes them promising candidates for optical amplification and down-conversion applications.

Although there are a few examples of star-shaped conjugated systems with the BODIPY unit attached to porphyrazine [25], subporphyrin [26] and truxene [27–28] cores via its *meso*-position by a phenylethynyl linkage, to the best of our knowledge there is no report on the synthesis of oligofluorene star-shaped systems with BODIPY as a core. The latter would provide extended conjugation not only through the β-position but via the pyrrole unit as well. It is known that attaching aromatic units to the β-positions of BODIPY increase the Stokes shift due to more pronounced structural relaxation of the excited state [29]. Therefore, incorporating the BODIPY unit as the core could improve the separation between absorption and emission spectra of the material and thereby decrease its self-absorption. We have recently shown among oligofluorene–BODIPY diads that compounds with oligofluorenes attached at the β-position of BODIPY have a significantly higher Stokes shift than those with oligofluorenes at the *meso*-position [30].

**Figure 1:**
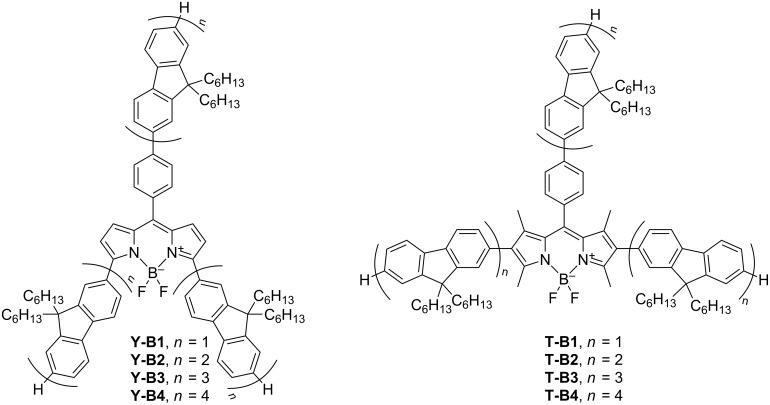
The structures of the star-shaped oligofluorenes with BODIPY cores, **Y-Bn** (*n* = 1–4) and **T-Bn** (*n* = 1–4).

In this paper we report the synthesis and physicochemical properties of two series of star-shaped oligofluorenes with a BODIPY core (Figure 1). In one of them (**Y-Bn**, *n* = 1–4) the oligofluorene arms are attached to the *meso*-position (via a phenylene linker) and α-positions of the central BODIPY unit, yielding Y-shaped molecules. The other series (**T-Bn**, *n* = 1–4) represents star-shaped systems with the arms in the *meso*- and β-positions, providing T-shaped molecules.

## Results and Discussion

### Synthesis

**Scheme 1:**
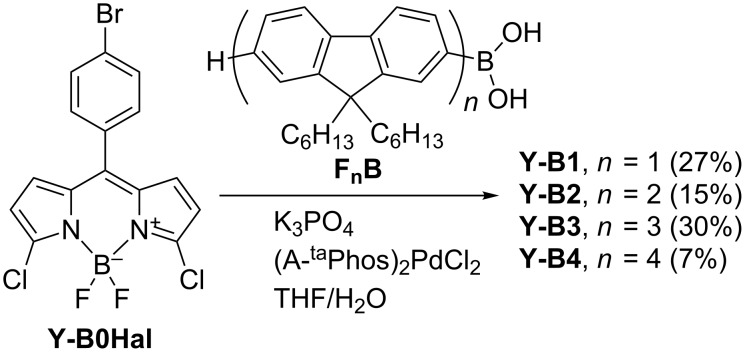
Synthesis of the **Y-Bn** (*n* = 1–4) series.

For the synthesis of the **Y-B1**–**Y-B4** series a convergent strategy was chosen (Scheme 1). The modified Suzuki coupling protocol with K_3_PO_4_ as a base was used to avoid decomposition of the core-precursor **Y-B0Hal** under strong basic conditions. To achieve maximum coupling efficiency with the chloro-substituted BODIPY core, the catalyst (A-^ta^Phos)_2_PdCl_2_ was applied, which was reported to have greater affinity towards heteroaryl chlorides [31]. The yields of the coupling reactions were poor (Scheme 1) and generally decreased with increasing length of the arm. Surprisingly, the highest yield (30%) was achieved for **Y-B3**. The compound **Y-B0Hal** was synthesised using a procedure reported previously [32]. The series of oligofluorenylboronic acids **F_n_B** (*n* = 1–4), synthesised by a known procedure [19], was used as the precursors for the nucleophilic coupling reagent.

**Scheme 2:**
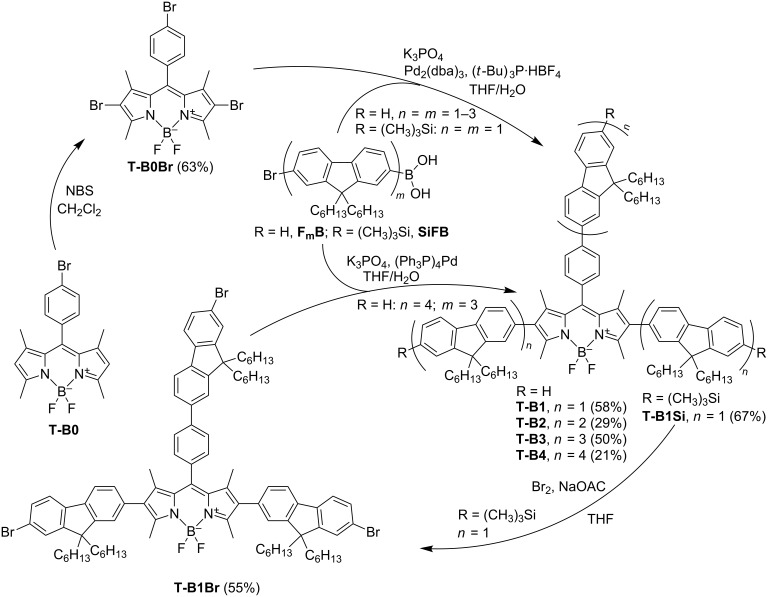
Synthesis of the **T-Bn** (*n* = 1–4) series.

In the case of the **T-Bn** series, the convergent strategy was used for the synthesis of **T-B1**–**T-B3** compounds using Suzuki coupling of the aforementioned oligofluorenyl boronic acids **F_n_B** (*n* = 1–3) with the core precursor compound **T-B0Br** [33], using conditions which proved to be efficient for coupling of BODIPY derivatives [30] (Scheme 2). The yields observed were 29–58%. Conversely, the **T-B4** member of the series was synthesised by a semi-convergent approach, firstly via Suzuki coupling of the brominated core **T-B0Br** with trimethylsilylfluorenylboronic acid **SiFB** (68% yield). Subsequent electrophilic ipso-substitution of tris(trimethylsilylfluorenylBODIPY) **T-B1Si** by molecular bromine, in the presence of a weak base (KOAc) to avoid acidic conditions, gave **T-B1Br** in 55% yield. Finally, Suzuki coupling of the bromo compound **T-B1Br** with terfluorenyl boronic acid **F_3_B** was achieved in 21% yield. The boronic acid **SiFB** was synthesised using a previously published procedure [23]. The core precursor **T-B0Br** was synthesised in 63% yield by using NBS as the brominating reagent instead of molecular bromine which was used previously [33].

### Thermal, electrochemical and optical properties

#### TGA and DSC analyses

All of the final target compounds showed good thermal stability. Thermogravimetric analysis (TGA) revealed decomposition temperatures in the range 396–442 °C (Table 1, Figure S1, Supporting Information File 1). The morphological stability was evaluated by differential scanning calorimetry (DSC). All of the synthesised compounds were found to be amorphous materials and exhibited reasonably high glass transition temperatures (*T*_g_) (Table 1, Figure S2, Supporting Information File 1). The *T*_g_ value generally increased with increasing molecular weight. This trend was most pronounced for **Y-Bn** (*n* = 1–4) and not so significant for the **T-Bn** (*n* = 1–4) series. In the latter case the *T*_g_ reached a saturation value at 99 °C for terfluorene **T-B3**.

**Table 1:** Thermal properties of oligofluorene–BODIPY compounds.

Compound	*M*_w_, Da	*T*_d_,^a^ °C	*T*_g_,^b^ °C

**Y-B1**	1265.63	438	63
**Y-B2**	2263.20	421	80
**Y-B3**	3260.76	442	87
**Y-B4**	4258.33	442	96
**T-B1**	1321.74	396	83
**T-B2**	2319.30	426	93
**T-B3**	3316.87	440	99
**T-B4**	4314.43	413	96

^a^*T*_d_: decomposition temperature corresponding to 5% mass loss (TGA was performed at 10 °C/min). ^b^DSC was performed at 10 °C/min.

#### Electrochemistry

The electrochemical properties of the materials were studied in dichloromethane solutions with the concentration of the analyte ca. 10^−4^ M, and the results are summarised in Table 2.

**Table 2:** Electrochemical data for oligofluorene–BODIPY compounds and HOMO–LUMO gaps calculated from cyclic voltammetry and electronic absorption spectroscopy.

Comp.	*E*_p_^ox^,^a^ vs Fc/Fc^+^, V	HOMO,^b^ eV	*E*_p_^red^,^c^ vs Fc/Fc^+^, V	LUMO,^b^ eV	*E*_g_, eV	
					CV^d^	UV^e^

**Y-B1**	0.69/0.63, 1.26	−5.38 (0.58)	−1.21/−1.07, −1.97	−3.72 (−1.08)	1.66	1.91 (648)
**Y-B2**	0.81/0.73, 1.11/1.02, 1.27/1.18, 1.44	−5.50 (0.70)	−1.48, −2.14	−3.64 (−1.16)	1.86	1.87 (664.5)
**Y-B3**	0.74/0.67, 0.96/0.88, 1.12/1.08, 1.21/1.14, 1.32, 1.53	−5.43 (0.63)	−1.62, −2.25	−3.54 (−1.26)	1.89	1.86 (665)
**Y-B4**	0.74/0.68, 0.89/0.83, 1.07/0.94, 1.32, 1.50	−5.42 (0.62)	−1.55, −2.20	−3.63 (−1.17)	1.79	1.86 (668)
**T-B1**	0.70/0.62, 1.23	−5.39 (0.59)	−1.94/−1.54	−3.34 (−1.46)	2.05	2.15 (576)
**T-B2**	0.68/0.63, 0.97/0.87, 1.35	−5.38 (0.58)	−1.77/−1.19	−3.31 (−1.49)	2.07	2.13 (582)
**T-B3**	0.84/0.78, 0.98/0.90, 1.24/1.12, 1.59	−5.52 (0.72)	−1.80/−1.17	−3.32 (−1.48)	2.20	2.13 (582)
**T-B4**	0.78/0.72, 0.88/0.82, 1.05/0.95, 1.42, 1.58	−5.48 (0.68)	−1.80/−1.09	−3.38 (−1.42)	2.10	2.13 (582)

^a^For a reversible and quasi-reversible oxidation wave, when peaks were observed on both direct and reversed scans, each wave is presented as anodic/cathodic peak values, otherwise only anodic peaks observed on the direct scan are presented. ^b^HOMO(LUMO) level is calculated by the formula *E*^HOMO(LUMO)^ = − (*E*_onset_^ox(red)^ + 4.80), *E*_onset_^ox(red)^ values are shown in the brackets. ^c^For a reversible and quasi-reversible reduction wave, when peaks were observed on both direct and reversed scans, each wave is presented as cathodic/anodic peak values, otherwise only cathodic peaks observed on the direct scan are presented. ^d^Electrochemical HOMO–LUMO gap is calculated as a difference between HOMO and LUMO levels. ^e^Optical HOMO–LUMO gap is calculated by the formula *E*_g_ = 1239.84/λ_onset_ from absorption onset (λ_onset_) shown in the brackets (in nm).

The redox properties of the materials were investigated by cyclic voltammetry and the oxidation and reduction waves of **Y-Bn** (*n* = 1–4) and **T-Bn** (*n* = 1–4) series are presented in Figures S3 and S4, respectively (Supporting Information File 1). The oxidation waves are positioned at similar potentials for the analogues of both families. For the first member of each series, **Y-B1** and **T-B1**, there are only two oxidation waves – the first is reversible (*E*_1/2_ = 0.66 V) and the other irreversible (*E*_pa_ > 1.2 V). The potential of the former is too low to be related to the oxidation of the arms [34] or an isolated BODIPY unit [35]. It is probably due to the oxidation of the fluorene–BODIPY–fluorene structural unit, with the fluorene arms attached in the α- and β-positions for **Y-B1** and **T-B1**, respectively. It is consistent with the result of DFT calculations which show the location of the HOMO on this unit (vide infra). This first oxidation waves have exactly the same half wave potential for **T-B2** but the values are slightly shifted for the other members of the two series (**Y-B2**–**Y-B4**, **T-B3** and **T-B4**), with *E*_1/2_ ranging from 0.71 to 0.81 V. The cyclic voltammetry of **Y-B2**–**Y-B4** and **T-B2**–**T-B4** reveals that the second oxidation waves in these compounds are reversible. This wave is more intense than the first one in all cases. It probably also relates to the oxidation of the oligofluorene–BODIPY–oligofluorene unit, but the larger peak current intensity indicates a greater involvement of the oligofluorene in the *meso*-position. The cyclic voltammetry experiments of **Y-B2**–**Y-B4**, **T-B3** and **T-B4** show a third quasi-reversible oxidation peak related to the formation of a radical trication on the oligofluorenes [34]. The second and third oxidation peaks appear at lower potentials in the larger analogues due to the longer effective conjugation length of the molecules. The irreversible peaks that the materials show between 1.30 V and 1.60 V are probably due to over-oxidation of the molecule.

All the oligomers of the **Y-Bn** (*n* = 1−4) series feature a reduction wave corresponding to the formation of a radical anion in the BODIPY core [36], but its position and reversibility varies for the different members of the family, being reversible and at a less negative potential for **Y-B1** (*E*_1/2_ = −1.14 V). Due to conjugation between the BODIPY core and the arms in its *α*-position, the electronic structure of **Y-B1** provides stabilisation of the negative doped states owing to partial delocalisation of charge/spin density over the unit α-fluorene–BODIPY–α-fluorene. This can be seen from DFT calculations for this structure (vide infra) which show slight expansion of the LUMO from the core to the arm. The increase in reduction potential with increasing length of the arm could be explained by the lower electron density exerted by one fluorene in each arm, as opposed to the oligofluorenes in the other members of the series. Due to the aforementioned conjugation, the increase in the oligofluorene arm length provides greater electron density to the core, which leads to more negative reduction potentials. The first reduction wave for the rest of the materials is quasi-reversible while the second one is irreversible for all the compounds. The second reduction wave corresponds to the formation of a dianion, most likely localised on the BODIPY core and partially delocalised on the arms.

In contrast to the **Y-Bn** (*n* = 1–4) series, the reduction of oligomers from the **T-Bn** (*n* = 1–4) family exhibits only one quasi-reversible wave in the case of **T-B1** or irreversible in the case of **T-Bn** (*n* = 2–4), with very similar cathodic peak potentials. The different reduction behaviour of **T-Bn** (*n* = 1–4) is attributed to the poorer conjugation between the core and the oligofluorene arms in the β-positions which is consistent with DFT calculations (vide infra), showing in this case localisation of the LUMO exclusively on the BODIPY core. The positive inductive effect of four methyl groups attached to the core can also contribute to a decrease in the electron affinity of the BODIPY unit in this case.

The HOMO levels for both families are similar, whereas, in general, the LUMO levels of the **Y-Bn** family are lower, leading to narrower electrochemical HOMO–LUMO gaps.

#### Optical properties

**Figure 2:**
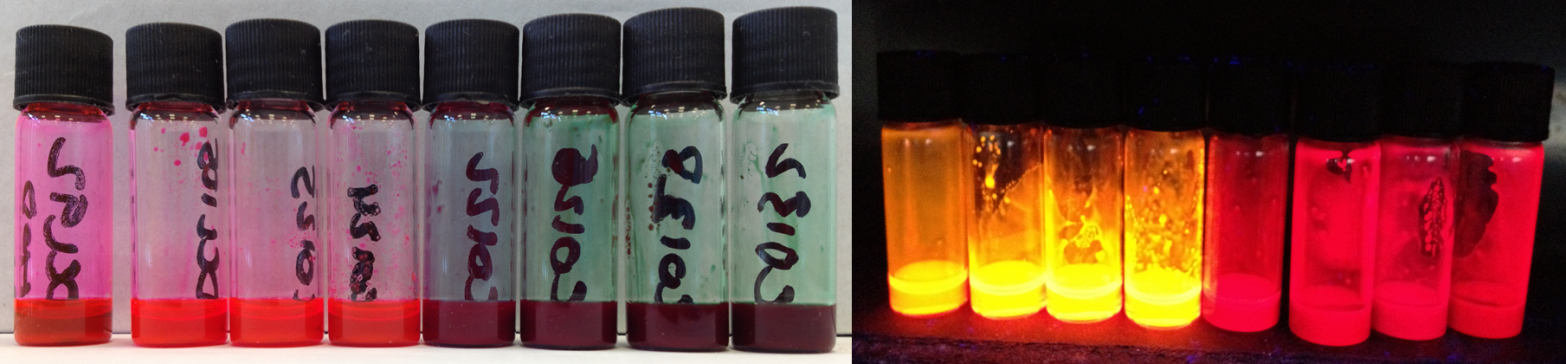
The samples of oligofluorene BODIPY solutions in toluene under ambient light (left) and UV illumination (254 nm, right). From left to right, **T-B1**, **T-B2**, **T-B3**, **T-B4**, **Y-B1**, **Y-B2**, **Y-B3** and **Y-B4**.

**Figure 3:**
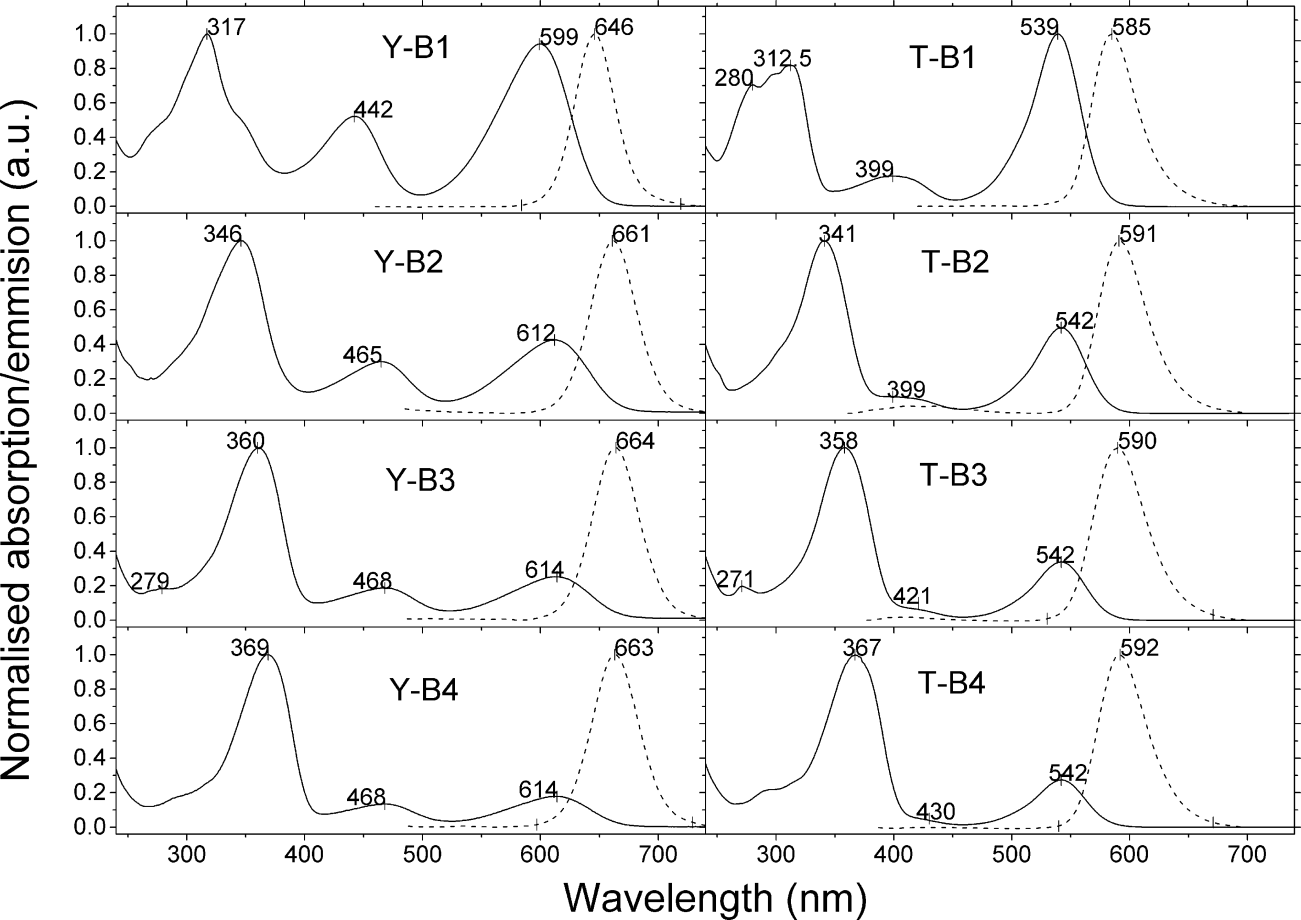
The normalised absorption (solid lines) and emission (dash lines) spectra of **Y-Bn** (*n* = 1–4) (left panel) and **T-Bn** (*n* = 1–4) (right panel) series.

The optical properties of the oligomers were studied in dichloromethane solutions. An image of toluene solutions of both families under ambient conditions and UV-illumination is presented in Figure 2. The effect of the substitution pattern of the BODIPY core with fluorene arms (either at the α- or β-positions) on the optical properties of the materials can be observed with the naked eye. The compounds of the **Y-Bn** (*n* = 1–4) series are dark green powders and exhibit a dark red colour in concentrated solutions and green when diluted. The colour of the **T-Bn** (*n* = 1–4) oligomers is bright pink, both in the powder form and in solution, and they give bright orange photoluminescence. The deep-red fluorescence of the **Y-Bn** (*n* = 1–4) series seems less efficient to the naked eye than that of the **T-Bn** family. The optical properties of the **T-Bn** and **Y-Bn** families are summarised in Table S1 (Supporting Information File 1) and Figure 3.

The absorption spectra of all the compounds exhibit an intense short-wave band associated with the π–π* transition of the oligofluorene arms [19]. As the number of fluorene units in the arm increases, this band becomes more intense and subjected to a bathochromic shift. The spectra of all compounds reveal the two features which are related to the presence of the BODIPY unit and can be assigned to S_0_–S_1_ and S_0_–S_2_ transitions [37]. The band around 400–470 nm is attributed to the S_0_–S_2_ transition and it is clearly resolved for oligomers of the **Y-Bn** (*n* = 1–4) series and in the spectrum of **T-B1**. In the spectra of the larger members of the **T-Bn** family the S_0_–S_2_ transition becomes a shoulder of the π–π* oligfluorene band as the latter red-shifts. The longest wavelength bands observed in the absorption spectra corresponds to the S_0_–S_1_ transition. These two bands were found to be more affected by the length of the arm in the case of **Y-Bn** (*n* = 1–4) series due to better conjugation between the core and the arms. The optical and electrochemical HOMO–LUMO gaps are in good agreement for most compounds, the difference being greatest for the first members of the two series **Y-B1** (0.25 eV) and **T-B1** (0.10 eV).

The emission spectra of the oligomers reveal a single band with its peak positioned at around 600 nm for the **T-Bn** compounds and 660 nm for the **Y-Bn** series. The emission originates from the BODIPY core which is an indication of an efficient energy transfer observed in these conjugated systems from the fluorene components to the core. A Stokes shift of around 50 nm was observed in both series, corresponding to 1230 cm^−1^ for **Y-Bn** and 1560 cm^−1^ for **T-Bn**. If one considers the efficient energy transfer from the oligofluorene arms to the BODIPY core, the separation between absorption and emission overall becomes much higher and the materials can therefore be used as efficient down-converters from UV to the red region of the spectrum.

#### DFT calculations

**Figure 4:**
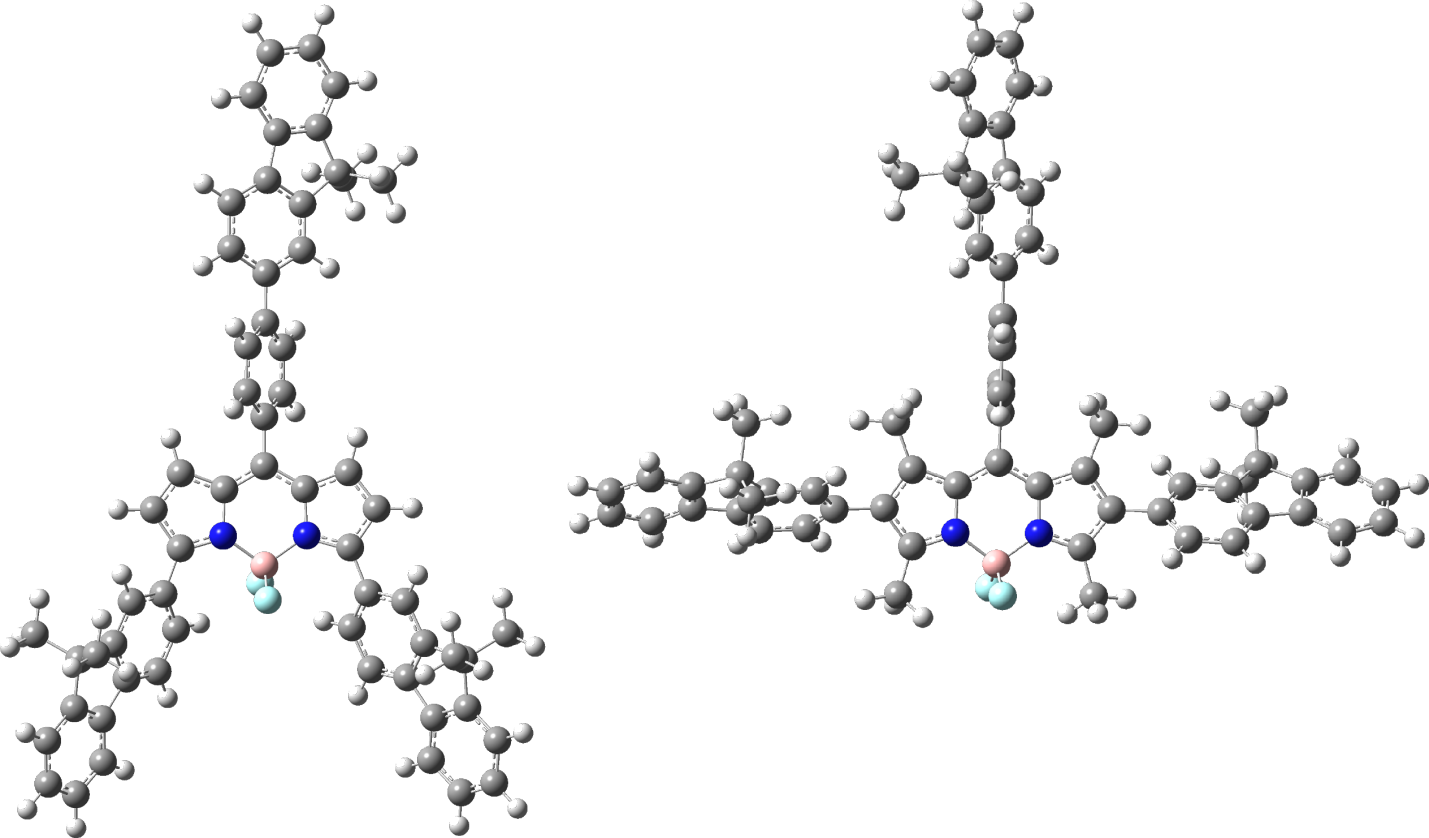
Optimised structures of **T-B1** (left) and **Y-B1** (right).

The structures of **T-B1** and **Y-B1** were optimised using the program Gaussian 09 [38] with the CAM-B3LYP [39] functional and the TZVP [40] basis set. Solvent effects were considered with the inclusion of the SMD [41] solvent model. In order to increase the computational efficiency of the optimisations, the hexyl chains on the fluorene units were shortened to methyl groups. The twist between the β-fluorenes and BODIPY in **T-B1** (56.6°) is greater than that obtained for the fluorenes in α-positions in **Y-B1** (42.9°), probably due to steric restrictions imposed by the methyl substituents at the 1,3,5,7-positions (Figure 4). The distance between the hydrogens of the fluorene and the fluorines of the BODIPY in **Y-B1** are 2.20 Å and 2.23 Å. These distances are shorter than the sum of the van der Waals radii of H (1.20 Å) and F (1.47 Å), which is 2.67 Å. This implies that there are H–F interactions between the fluorene and the BODIPY core, which could also contribute to stabilise the planarisation of the structure. In the case of **T-B1**, the H–F distances between the hydrogens of the methyl substituents at the 3,5-positions and the fluorines at the 4-position of the BODIPY (2.42 Å and 2.44 Å) are also smaller than the sum of the van der Waals radii, also indicating the possibility of H–F interactions. The greater degree of planarity in **Y-B1** leads to increased conjugation and therefore a lower HOMO–LUMO gap, which is predicted from the DFT calculations and is observed in the electrochemical and optical data. This explains the red shifts in the absorption spectrum of **Y-B1** compared to that of **T-B1** (Δλ = 60 nm). It is likely that the greater degree of planarisation is also present in the rest of the members of the **Y-Bn** family, which would account for their bathochromically shifted optical transitions compared with their T-shaped analogues. The phenyl linker attached to the *meso*-position is also more twisted in **T-B1** (80.8°) than in **Y-B1** (58.0°) because the methyl groups in the 1,7-positions exert greater steric hindrance than the hydrogen substituents of **Y-B1**.

**Figure 5:**
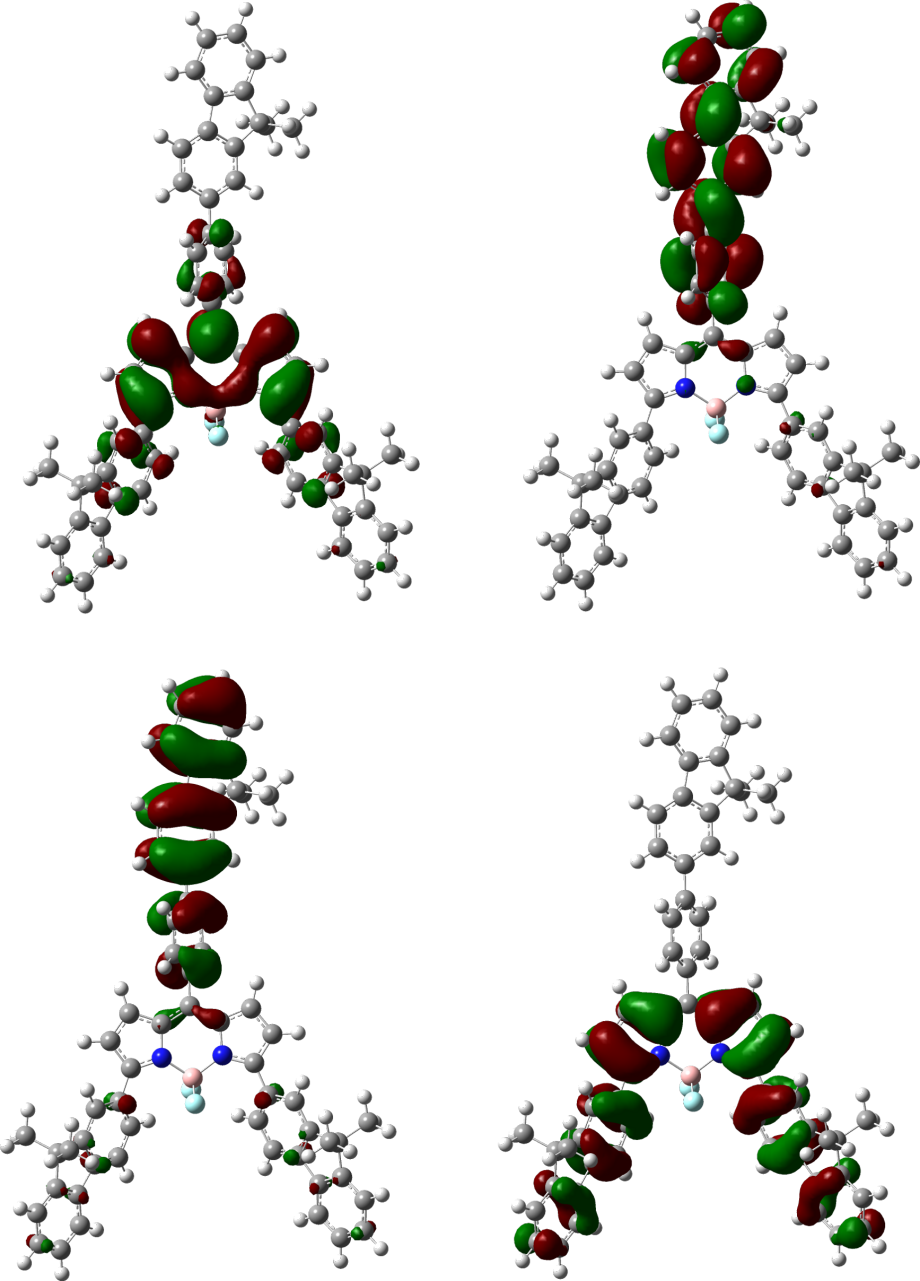
HOMO−1 (bottom, left), HOMO (bottom, right), LUMO (top, left) and LUMO+1 (top, right) of **Y-B1**.

**Figure 6:**
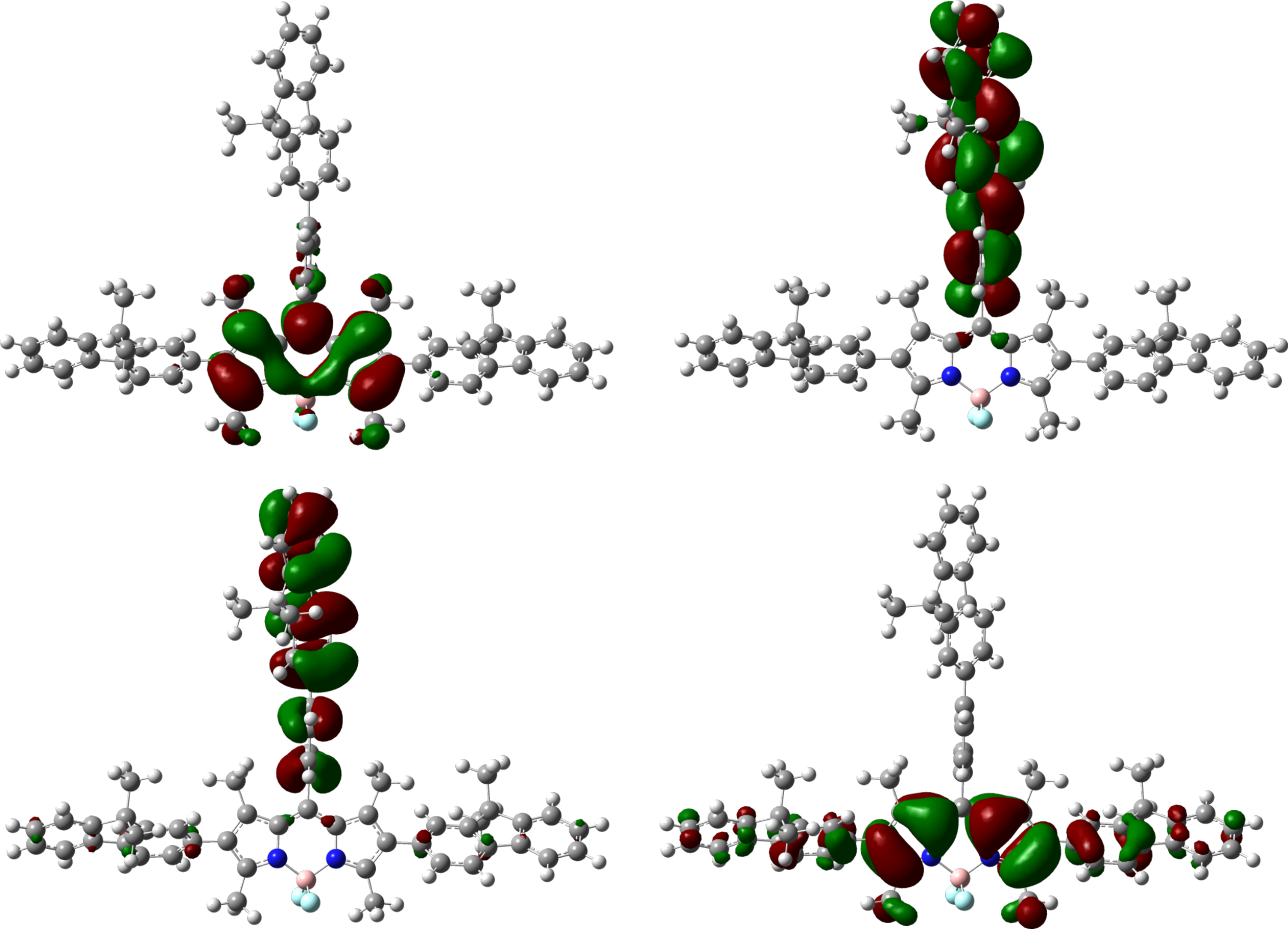
HOMO−1 (bottom, left), HOMO (bottom, right), LUMO (top, left) and LUMO+1 (top, right) of **T-B1**.

The greater degree of conjugation of the fluorenes with the core in **Y-B1** compared with **T-B1** is also evident from the examination of their frontier orbitals. The HOMO of **Y-B1** shows increased electron-density over the fluorene units (Figure 5) with respect to the analogous units in **T-B1** (Figure 6). Also, the LUMO of **Y-B1** shows some delocalisation over the fluorenes, but the LUMO of **T-B1** is localised on the BODIPY unit, which supports the idea that the increase in the fluorene twist reduces conjugation in **T-B1**, hence its absorption spectrum is hypsochromically shifted with respect to **Y-B1**. The calculated energies of the HOMO and LUMO levels do not match the experimental values but they show the trend of lower HOMO–LUMO gaps for **Y-B1**.

The HOMO−1 and the LUMO+1 frontier orbitals are localised primarily over the phenyl-fluorene located on the *meso*-position for both compounds and they have almost the same calculated energies. The absorption band that occurs in the region of 400–470 nm has previously been described as the S_0_–S_2_ transition of the BODIPY unit [37].

The TDDFT results (see Supporting Information File 1, Table S2) show the lowest energy peak for each compound studied, which represents a transition from HOMO to LUMO. The bathochromic shift of the peak of **Y-B1** with respect to that of **T-B1** is explained by the smaller twist of the fluorenes in the α-position in **Y-B1** which results in some of the electron density in the LUMO being located on the fluorene units which is not the case for **T-B1**. Likewise, the transitions from HOMO−2 and HOMO−1 to the LUMO mainly contribute to the second peak, with the more delocalised LUMO of **Y-B1** leading to a red-shifted peak.

## Conclusion

Here, we have presented the synthesis and physicochemical study of two novel series of star-shaped conjugated systems with BODIPY cores and oligofluorene arms. All the oligomers were found to be amorphous solids with reasonably high glass transition temperatures. The conjugated systems studied showed efficient energy transfer with good separation between absorption and emission profiles. Their efficient down-conversion efficiency has been studied very recently in devices for visible light communications, showing great potential to replace commercially available phosphor-based materials in light emitting diodes [42]. In summary, incorporation of BODIPY as a core in star-shaped oligofluorene systems was shown to be useful in providing amorphous materials for photonic applications with high Stokes shifts and low levels of self-absorption.

## Experimental

### General experimental

Unless otherwise stated, all reagents and solvents were purchased commercially from Sigma-Aldrich or Alfa Aesar and were used without any purification. Dry solvents (dichloromethane, tetrahydrofuran, toluene, hexane and diethyl ether) were obtained from a solvent purification system (SPS 400, innovative technologies) using alumina as the drying agent; any other dry solvents were purchased from Sigma-Aldrich. ^1^H and ^13^C NMR spectra were recorded on a Bruker Avance DPX400 at 400.13 and 100.61 MHz or a Bruker Avance DRX500 at 500 MHz and 125.75 MHz in CDCl_3_ or CD_2_Cl_2_. Proton NMR chemical shifts are reported as δ values in ppm. The chemical shifts were calibrated using reported values for residual solvent signals [43] for ^1^H NMR: 7.26 (CDCl_3_) or 5.32 (CD_2_Cl_2_); for ^13^C NMR: 77.16 (CDCl_3_) or 53.84 (CD_2_Cl_2_). Data are presented as follows: chemical shift, number of nuclei based on integration, multiplicity (s = singlet, d = doublet, dd = doublet of doublets, m = multiplet), and coupling constant(s) (^3^*J or ^4^J*) are in Hz. Multiplets are reported over the range (in ppm) in which they appear.

MS MALDI–TOF spectra were recorded on a Shimadzu Axima-CFR spectrometer (mass range 1–150,000 Da). Retinoic acid was used as a matrix. Elemental analyses were obtained on a PERKIN ELMER 2400 elemental analyser. Commercial TLC plates (Silica gel 60 F254) were used for TLC chromatography and column chromatography was carried out on silica gel Zeoprep 60 (40–63 µm). Solvents were removed using a rotary evaporator (vacuum supplied by low vacuum pump) and, when necessary, a high vacuum pump was used to remove residual solvent.

Thermogravimetric analysis (TGA) was performed using a Perkin-Elmer Thermogravimetric Analyser TGA7 under a constant flow of argon. Differential Scanning Calorimetry (DSC) was conducted on a TA Instruments Q1000 with a RC-90 refrigerated cooling unit attached. The calibration was conducted using indium (melt temperature 156.42 °C, ∆*H*_f_ 28.42 J/g). The test procedure used was a standard heat-cool-reheat cycle, which allows the removal of thermal history on the first heat allowing examination of any thermal processes on the cooling and second heat scan. The temperature range was from −50 °C to 300 °C at 10 °C/min.

Cyclic voltammetry measurements were performed on a CH Instruments 660A electrochemical workstation with *iR* compensation using anhydrous dichloromethane as a solvent. The electrodes were glassy carbon, platinum wire, and silver wire as the working, counter, and reference electrodes, respectively. All solutions were degassed (Ar) and contained monomer substrates in concentrations of ca. 10^−4^ M, together with TBAPF_6_ (0.1 M) as the supporting electrolyte. All measurements are referenced against the *E*_1/2_ of the Fc/Fc^+^ redox couple.

UV–vis absorption spectra were recorded on a UNICAM UV 300, a Jasco V-660 or a Shimazdu UV-2600 spectrophotometer. Baselines of solvents were measured before analysis and solution spectra were recorded in 1 cm or 1 mm path length quartz cells between 190 and 900 nm. Emission spectra were measured on a Perkin Elmer LS45 or a Jasco FP-6500 fluorescence spectrometers.

## Supporting Information

File 1 Experimental procedures for all new compounds, thermal analysis, cyclic voltammograms and associated data, photophysical data, computational data, ^1^H NMR spectra for all new compounds.
