# Supplementary material for: Synthesis and properties of novel star-shaped oligofluorene conjugated systems with BODIPY cores
Source: Beilstein J Org Chem. 2014 Nov 19;10:2704–14. doi: 10.3762/bjoc.10.285 (PMC4273294; doi:10.3762/bjoc.10.285)
Supplement: File 1 — Experimental procedures for all new compounds, thermal analysis, cyclic voltammograms and associated data, photophysical data, computational data, 1H NMR spectra for all new compounds. [file Beilstein_J_Org_Chem-10-2704-s001.pdf]

Supporting Information

for

Synthesis and properties of novel star-shaped oligofluorene conjugated systems with BODIPY cores

Clara Orofino-Pena[1], Diego Cortizo-Lacalle[1], Joseph Cameron[1], Muhammad T. Sajjad[2], Pavlos P. Manousiadis[2], Neil J. Findlay[1], Alexander L. Kanibolotsky[1,3], Dimali Amarasinghe[2], Peter J. Skabara[*1], Tell Tuttle[1], Graham A. Turnbull[*2] and Ifor D. W. Samuel[2]

Address: [1]WestCHEM, Department of Pure and Applied Chemistry, University of Strathclyde, Glasgow, G1 1XL, UK, [2]Organic Semiconductor Centre, SUPA, School of Physics & Astronomy, University of St. Andrews, St. Andrews, KY16 9SS, UK and [3]Institute of Physical-Organic Chemistry and Coal Chemistry, 83114 Donetsk, Ukraine

Email: Peter J. Skabara[*] - peter.skabara@strath.ac.uk; Graham A. Turnbull[*] - gat@st-andrews.ac.uk

* Corresponding author

Experimental procedures for all new compounds, thermal analysis, cyclic voltammograms and associated data, photophysical data, computational data, ^{1}H NMR spectra for all new compounds

2,8-Dibromo-10-(4-bromophenyl)-5,5-difluoro-1,3,7,9-tetramethyl-5*H*-dipyrrolo[1,2-*c*:2',1'-*f*][1,3,2]diazaborinin-4-ium-5-uide (**T-B0Br**)

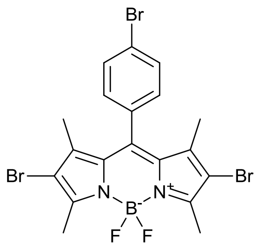

10-(4-Bromophenyl)-5,5-difluoro-1,3,7,9-tetramethyl-5*H*-dipyrrolo[1,2-*c*:2',1'-*f*][1,3,2]diazaborinin-4-ium-5-uide [1] (**TB0**) (500 mg, $1.24 \cdot 10^{-3}$ mol) was dissolved in dry dichloromethane (20 mL) and the solution was protected from the light. *N*-Bromosuccinimide (596 mg, 3.35 mmol) was quickly added and the reaction mixture was stirred for 18 hours under nitrogen. The reaction was quenched with water. The organic phase was separated and the aqueous layer extracted with further portions of CH_2Cl_2. The combined extracts were washed with water, dried over $MgSO_4$, and the solvent evaporated. The crude product was purified by column chromatography on silica gel eluting with hexane:dichloromethane (7:3). The product was obtained as a red solid (441 mg, 0.786·mmol, 63%). m.p. 133-135 °C. ^1H NMR ($CDCl_3$, δ, *400* MHz): 7.69 (2 H, d, ^3J = 8.4 Hz), 7.19 (2 H, d, ^3J = 8.4 Hz), 2.56 (6 H, s), 1.41 (6 H, s). ^{13}C NMR ($CDCl_3$, δ, *100* MHz): 154.32, 140.84, 140.69, 133.36, 132.92, 132.89, 130.31, 129.90, 129.87, 129.84, 123.96, 111.91, 13.86, 13.58. (MALDI/TOF, m/z): [M^+] calcd. for $C_{19}H_{16}BBr_3F_2N_2$: 560.9; found, 559.8. The spectra are similar to the ones described in the literature [2].

General procedure A: Synthesis of T-shaped tris(oligofluorene) BODIPYs by Suzuki cross-coupling (**T-B1**, **T-B1Si**, **T-B2**, **T-B3**)

2,8-Dibromo-10-(4-bromophenyl)-5,5-difluoro-1,3,7,9-tetramethyl-5*H*-dipyrrolo[1,2-*c*:2',1'-*f*][1,3,2]diazaborinin-4-ium-5-uide (**T-B0Br**) (1 equiv), (oligo(9,9'-dihexylfluoren)-2-yl)boronic acid (**F_nB** (n = 1–3) or **SiFB**) (1.2 equiv), $Pd_2(dba)_3$ (0.03 equiv) and $P(t\text{-Bu})_3HBF_4$ (2 equiv per Pd catalyst) were dissolved in dry tetrahydrofuran (20 mL). An aqueous solution of K_3PO_4 (1.44 M, 1.20 equiv) was added to the previous solution and the reaction mixture was degassed and heated up to 70 °C. The reaction was refluxed under nitrogen for 48 h. It was then dissolved in dichloromethane (50 mL) and washed with water (3 x 50 mL). The organic layer was dried over $MgSO_4$, filtered and the solvents evaporated under reduced pressure. The crude product was purified by column chromatography on silica gel eluting with hexane:dichloromethane. The product was dissolved in the minimum amount of dichloromethane and precipitated from methanol to yield the product.

2,8-Bis(9,9-dihexyl-9*H*-fluoren-2-yl)-10-(4-(9,9-dihexyl-9*H*-fluoren-2-yl)phenyl)-5,5-difluoro-1,3,7,9-tetramethyl-5*H*-dipyrrolo[1,2-*c*:2',1'-*f*][1,3,2]diazaborinin-4-ium-5-uide (**T-B1**)

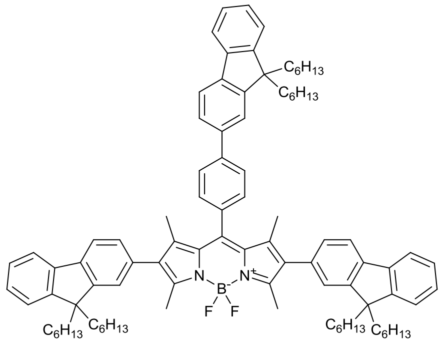

Using the general procedure A, the quantities used were: (**T-B0Br**) (88 mg, 0.16 mmol), 9,9'-dihexyl-9*H*-fluoren-2-yl)boronic acid (**F_1B**) (210 mg, 0.555 mmol), $Pd_2(dba)_3$ (20 mg, 0.022 mmol), P(*t*-Bu)$_3$·HBF_4 (36 mg, 0.12 mmol), K_3PO_4 1.44 M (0.50 mL, 0.72 mmol). Column hexane:dichloromethane (3:1). The product was obtained as an intense pink powder (120 mg, 0.091·mmol, 58%). ^{1}H NMR ($CDCl_3$, δ, 400 MHz): 7.86 (2 H, d, ^3J = 8.0 Hz), 7.77 (1 H, d, ^3J = 8.0 Hz), 7.75-7.68 (5 H, m), 7.67-7.61 (2 H, m), 7.51 (2 H, d, ^3J = 8.4 Hz), 7.39-7.27 (9 H, m), 7.20-7.12 (4 H, m), 2.62 (6 H, s), 2.10-1.90 (12 H, m), 1.51 (6 H, s), 1.15-0.95 (36 H, m), 0.79-0.68 (30 H, m). ^{13}C NMR ($CDCl_3$, δ, 100 MHz): 154.53, 151.79, 151.08, 150.98, 150.90, 142.39, 141.98, 141.20, 140.92, 140.65, 140.23, 139.14, 138.84, 134.54, 134.34, 132.33, 131.62, 128.87, 128.78, 127.89, 127.39, 127.21, 126.98, 126.93, 126.11, 124.88, 123.09, 122.97, 121.22, 120.17, 119.99, 119.80, 119.63, 55.36, 55.20, 40.48, 31.60, 29.82, 29.74, 23.87, 22.69, 22.63, 14.11, 13.65, 13.37. (MALDI/TOF, m/z): [M^+] calcd. for $C_{94}H_{115}BF_2N_2$: 1321.7; found, 1321.6.

2,8-Bis(9,9-dihexyl-7-(trimethylsilyl)-9*H*-fluoren-2-yl)-10-(4-(9,9-dihexyl-7-(trimethylsilyl)-9*H*-fluoren-2-yl)phenyl)-5,5-difluoro-1,3,7,9-tetramethyl-5*H*-dipyrrolo[1,2-*c*:2',1'-*f*][1,3,2]diazaborinin-4-ium-5-uide (**T-B1Si**)

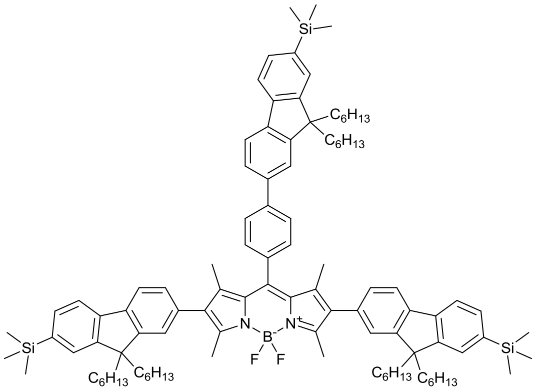

Using the general procedure A, the quantities used were: (**T-B0Br**) (260 mg, 0.464 mmol), (9,9'-dihexyl-7-(trimethylsilyl)-9*H*-fluoren-2yl)boronic acid (**SiF$_1$B**) (835 mg, 1.85 mmol), $Pd_2(dba)_3$ (40 mg, 0.044 mmol), $P(t\text{-}Bu)_3\cdot HBF_4$ (50 mg, 0.17 mmol), K_3PO_4 1.44 M (1.6 mL, 2.3 mmol). Column chromatography was performed using hexane:dichloromethane (5:3). The product was dissolved in the minimum amount of dichloromethane and precipitated from methanol to yield an intense pink powder (478 mg, 0.311 mmol, 67%). ^{1}H NMR ($CDCl_3$, δ, 500 MHz): 7.85 (2 H, d, ^3J = 8.5 Hz), 7.77 (1 H, d, ^3J = 8.0 Hz), 7.74-7.69 (3 H, m), 7.67 (2 H, d, ^3J = 7.5 Hz), 7.65-7.60 (2 H, m), 7.54-7.43 (8 H, m), 7.18-7.12 (4 H, m), 2.61 (6 H, s), 2.08-1.88 (12 H, m), 1.49 (6 H, s), 1.14-0.95 (36 H, m), 0.78-0.58 (30 H, m), 0.312 (9 H, s), 0.305 (18 H, m). ^{13}C NMR (CD_2Cl_2, δ, 100 MHz): 154.67, 152.27, 151.48, 150.55, 150.49, 142.69, 142.56, 141.84, 141.60, 141.49, 140.57, 139.92, 139.67, 139.38, 134.86, 134.56, 132.82, 132.31, 131.90, 129.17, 129.13, 128.18, 128.10, 126.37, 125.40, 121.68, 120.52, 120.00, 119.47, 119.30, 55.64, 55.47, 40.49, 31.81, 29.91, 24.15, 22.87, 22.83, 14.14, 13.71, 13.49. (MALDI/TOF, m/z): [M$^+$] calcd. for $C_{103}H_{139}BF_2N_2Si_3$: 1538.3; 1537.4.

5,5-Difluoro-1,3,7,9-tetramethyl-2,8-bis(9,9,9',9'-tetrahexyl-9*H*,9'*H*-[2,2'-bifluoren]-7-yl)-10-(4-(9,9,9',9'-tetrahexyl-9*H*,9'*H*-[2,2'-bifluoren]-7-yl)phenyl)-5*H*-dipyrrolo[1,2-*c*:2',1'-*f*][1,3,2]diazaborinin-4-ium-5-uide (**T-B2**)

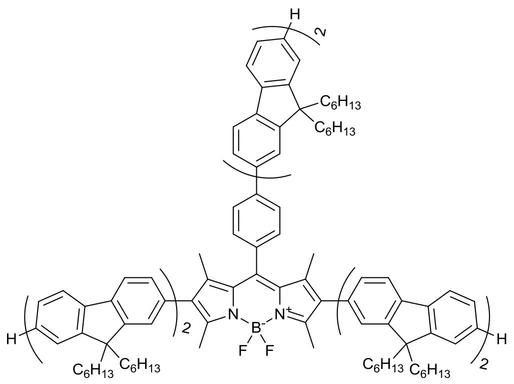

Using the general procedure A, the quantities used were: (**T-B0Br**) (90 mg, 0.16 mmol), (9,9,9',9'-tetrahexyl-9*H*,9'*H*-2,2'-bifluoren-7-yl)boronic acid (**F_2B**) (513 mg, 0.722 mmol), $Pd_2(dba)_3$ (20 mg, 0.022 mmol), P(*t*-Bu)$_3$·HBF_4 (36 mg, 0.12 mmol), K_3PO_4 1.44 M (0.60 mL, 0.86 mmol). Column chromatography was performed using hexane:dichloromethane (7:3). The product was obtained as an intense pink powder (108 mg, 0.0466·mmol, 29%). ^{1}H NMR ($CDCl_3$, δ, 400 MHz): 7.89 (2 H, d, ^3J = 8.4 Hz), 7.85-7.71 (12 H, m), 7.71-7.58 (14 H, m), 7.53 (2 H, d, ^3J = 8.4 Hz), 7.40-7.28 (9 H, m), 7.23-7.17 (4 H, m), 2.65 (6 H, s), 2.18-1.97 (24 H, m), 1.53 (6 H, s) (the singlet coincides with the water signal), 1.20-1.00 (72 H, m), 0.85-0.65 (60 H, m). ^{13}C NMR ($CDCl_3$, δ, 100 MHz): 154.56, 152.11, 151.75, 151.60, 151.21, 151.12, 142.70, 142.38, 140.91, 140.73, 140.53, 140.49, 140.46, 140.09, 139.94, 139.80, 139.15, 138.82, 134.78, 134.53, 132.32, 131.66, 128.98, 128.81, 127.90, 127.13, 126.92, 126.34, 126.27, 126.23, 126.15, 124.96, 123.06, 121.66, 121.55, 121.50, 120.25, 120.04, 120.02, 119.86, 119.72, 55.51, 55.35, 55.29, 40.49, 31.60, 31.57, 29.83, 29.73, 23.90, 22.70, 22.63, 14.15, 13.70, 13.42. (MALDI/TOF, m/z): [M^+] calcd. for $C_{169}H_{211}BF_2N_2$: 2319.30; found, 2318.19.

5,5-Difluoro-2,8-bis(9,9,9',9',9'',9''-hexahexyl-9*H*,9'*H*,9''*H*-[2,2':7',2''-terfluoren]-7-yl)-10-(4-(9,9,9',9',9'',9''-hexahexyl-9*H*,9'*H*,9''*H*-[2,2':7',2''-terfluoren]-7-yl)phenyl)-1,3,7,9-tetramethyl-5*H*-dipyrrolo[1,2-*c*:2',1'-*f*][1,3,2]diazaborinin-4-ium-5-uide (**T-B3**)

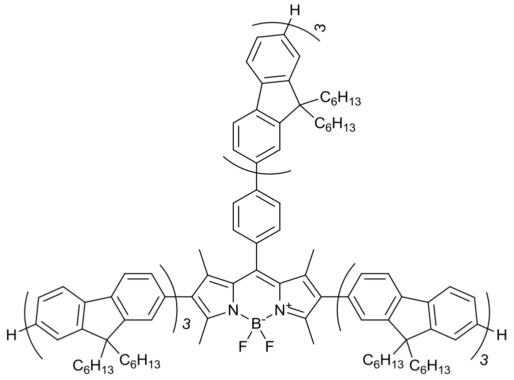

Using the general procedure A, the quantities used were: (**T-B0Br**) (100 mg, 0.178 mmol), (9,9,9',9',9'',9''-hexahexyl-9*H*,9'*H*,9''*H*,9'''*H*-2,2':7',2''-terfluoren-7-yl)boronic acid (**F$_3$B**) (887 mg, 0.85 mmol), $Pd_2(dba)_3$ (49 mg, 0.054 mmol), P(*t*-Bu)$_3$·HBF_4 (31 mg, 0.11 mmol), K_3PO_4 1.44 M (0.88 mL, 1.3 mmol). Column chromatography was performed using hexane:dichloromethane (7:3). The product was obtained as an intense pink powder (295 mg, 0.0889 mmol, 50%). ^{1}H NMR (CD_2Cl_2, δ, 400 MHz): 7.94 (2 H, d, ^3J = 8.0 Hz), 7.88-7.78 (15 H, m), 7.78-7.65 (29 H, m), 7.58 (2 H, d, ^3J = 8.0 Hz), 7.43-7.29 (9 H, m), 7.27-7.20 (4 H, m), 2.62 (6 H, s), 2.25-1.95 (36 H, m), 1.57 (6 H, s), 1.20-1.00 (108 H, m), 0.85-0.60 (90 H, m). ^{13}C NMR (CD_2Cl_2, δ, 100 MHz): 154.74, 152.41, 152.27, 152.19, 151.96, 151.63, 151.49, 142.71, 142.60, 141.25, 141.20, 141.02, 140.98, 140.90, 140.84, 140.58, 140.54, 140.47, 140.44, 140.31, 139.71, 139.28, 134.89, 134.62, 132.76, 131.96, 129.30, 129.24, 128.22, 127.46, 127.23, 126.52, 126.40, 125.42, 123.43, 121.96, 121.91, 121.68, 120.53, 120.38, 120.28, 120.09, 120.02, 55.91, 55.85, 55.74, 55.66, 40.77, 31.93, 30.09, 30.07, 30.03, 24.33, 24.29, 22.98, 22.92, 14.19, 13.76, 13.56. (MALDI/TOF, m/z): [M$^+$] calcd. for $C_{244}H_{307}BF_2N_2$: 3316.9; found 3316.2.

2,8-Bis(7-bromo-9,9-dihexyl-9*H*-fluoren-2-yl)-10-(4-(7-bromo-9,9-dihexyl-9*H*-fluoren-2-yl)phenyl)-5,5-difluoro-1,3,7,9-tetramethyl-5*H*-dipyrrolo[1,2-*c*:2',1'-*f*][1,3,2]diazaborinin-4-ium-5-uide (**T-B1Br**)

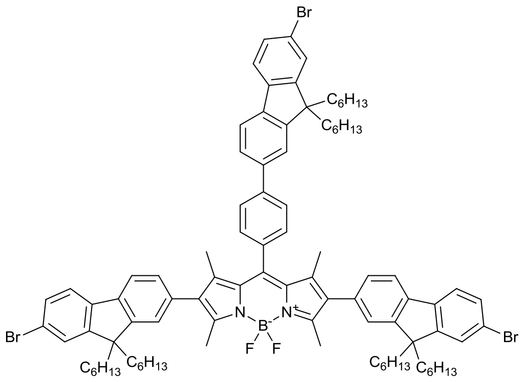

2,8-Bis(9,9-dihexyl-7-(trimethylsilyl)-9*H*-fluoren-2-yl)-10-(4-(9,9-dihexyl-7-(trimethylsilyl)-9*H*-fluoren-2-yl)phenyl)-5,5-difluoro-1,3,7,9-tetramethyl-5*H*-dipyrrolo[1,2-*c*:2',1'-*f*][1,3,2]diazaborinin-4-ium-5-uide (**T-B1TMS**) (400 mg, 0.26 mmol) and sodium acetate (66 mg, 0.8·mmol) were dissolved in dry tetrahydrofuran (16 mL). The solution was covered to exclude light and cooled to 0 ºC. A solution of bromine in dichloromethane (1.94 M, 1.3 mL, 2.5 mmol) was added to the previous solution. The reaction was stirred in the dark at 0 ºC for 30 min. The reaction was quenched with an aqueous saturated sodium sulphite solution and the mixture was dissolved in dichloromethane. The solvents were evaporated and the product was purified by column chromatography on silica gel eluting with hexane:dichloromethane (7:3). The product was dissolved in the minimum amount of dichloromethane and precipitated from methanol to yield a bright pink solid (224 mg, 0.144·mmol, 55%). ^{1}H NMR (CD_2Cl_2, *δ*, 400 MHz): 7.88 (2 H, d, ^3J = 8.4 Hz), 7.77 (1 H, d, ^3J = 8.0 Hz), 7.75-7.70 (2 H, m), 7.70-7.64 (2 H, m), 7.64-7.57 (3 H, m), 7.56-7.44 (8 H, m), 7.22-7.15 (4 H, m), 2.57 (6 H, s), 2.10-1.88 (12 H, m), 1.50[i] (6 H, s), 1.15-0.95 (36 H, m), 0.78-0.69 (18 H, m), 0.69-0.55 (12 H, m). ^{13}C NMR (CD_2Cl_2, *δ*, 100 MHz): 154.68, 153.75, 153.70, 151.73, 150.96, 142.58, 142.53,

140.41, 140.17, 139.74, 139.69, 139.52, 134.69, 134.66, 133.24, 131.89, 130.43, 130.37, 129.39, 129.20, 128.21, 126.75, 126.70, 126.62, 125.34, 121.62, 121.47, 121.41, 120.58, 120.07, 56.08, 55.90, 40.68, 40.59, 31.89, 30.00, 29.93, 24.17, 22.95, 22.89, 22.86, 14.14, 13.70, 13.47. (MALDI/TOF, m/z): [M^+] calcd. for $C_{94}H_{112}BBr_3F_2N_2$: 1558.4; found, 1558.2.

5,5-Difluoro-2,8-bis(9,9,9',9',9'',9'',9''',9'''-octahexyl-9*H*,9'*H*,9''*H*,9*H*'''-[2,2':7',2'':7'',2'''-quaterfluoren]-7-yl)-10-(4-(9,9,9',9',9'',9'',9''',9'''-octahexyl-9*H*,9'*H*,9''*H*,9'''*H*-[2,2':7',2'':7'',2'''-quaterfluoren]-7-yl)phenyl)-1,3,7,9-tetramethyl-5*H*-dipyrrolo[1,2-*c*:2',1'-*f*][1,3,2]diazaborinin-4-ium-5-uide (**T-B4**)

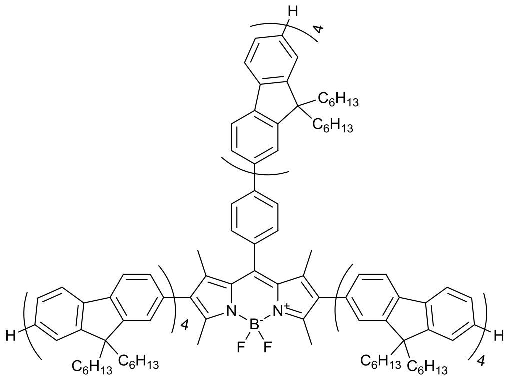

2,8-Bis(7-bromo-9,9-dihexyl-9*H*-fluoren-2-yl)-10-(4-(7-bromo-9,9-dihexyl-9*H*-fluoren-2-yl)phenyl)-5,5-difluoro-1,3,7,9-tetramethyl-5*H*-dipyrrolo[1,2-*c*:2',1'-*f*][1,3,2]diazaborinin-4-ium-5-uide (**T-B1Br**) (100 mg, 0.0642 mmol), (9,9,9',9',9'',9''-hexahexyl-9*H*,9'*H*,9''*H*,9'''*H* -2,2':7',2''-terfluoren-7-yl)boronic acid (**F_3B**) (311 mg, 0.298 mmol) and $Pd(PPh_3)_4$ (22 mg, 0.019 mmol) were dissolved in dry tetrahydrofuran (20 mL). An aqueous solution of K_3PO_4 (1.44 M, 0.64 mL, 0.922 mmol) was added to the previous solution and the reaction mixture was degassed and heated up to 70 °C. The reaction was refluxed under nitrogen for 3 days. The reaction mixture was quenched with water, diluted in dichloromethane and washed with water and brine. The organic layer was dried over $MgSO_4$, filtered and

the solvents evaporated under reduced pressure. The crude product was purified by column chromatography on silica gel eluting with hexane:dichloromethane (7:3). The product was dissolved in the minimum amount of dichloromethane and precipitated from methanol to yield an intense pink powder (59 mg, 0.0014 mmol, 21%). ^{1}H NMR (CD_2Cl_2, δ, 400 MHz): 7.94 (2 H, d, ^{3}J = 8.0 Hz), 7.89-7.78 (21 H, m), 7.78-7.65 (41 H, m), 7.58 (2 H, d, ^{3}J = 8.0 Hz), 7.43-7.30 (9 H, m), 7.28-7.18 (4 H, m), 2.62 (6 H, s), 2.30-1.90 (48 H, m), 1.57 (6 H, s), 1.20-1.00 (144 H, m), 0.85-0.60 (120 H, m). ^{13}C NMR (CD_2Cl_2, δ, 100 MHz): 154.75, 152.30, 152.21, 151.97, 151.64, 151.50, 142.72, 142.60, 141.26, 140.99, 140.93, 140.84, 140.54, 140.49, 140.32, 139.72, 139.29, 134.89, 134.64, 132.77, 131.97, 129.24, 128.25, 127.48, 127.24, 126.54, 126.42, 125.43, 123.44, 121.97, 121.68, 120.54, 120.40, 120.30, 120.10, 55.92, 55.86, 55.75, 55.67, 40.79, 31.94, 30.09, 30.04, 24.35, 24.30, 22.99, 22.93, 14.21, 13.78, 13.57. (MALDI/TOF, m/z): [M^{+}] calcd. for $C_{319}H_{403}BF_2N_2$: 4314.4; found 4314.9.

General procedure B: Synthesis of Y-shaped tris(oligofluorene) BODIPYs by Suzuki cross-coupling (**Y-B1, Y-B2, Y-B3, Y-B4**)

10-(4-Bromophenyl)-3,7-dichloro-5,5-difluoro-5*H*-dipyrrolo[1,2-*c*:2',1'-*f*][1,3,2]diazaborinin-4-ium-5-uide (**Y-B0Hal**) (1 equiv), (oligo(9,9’-dihexylfluoren) -2-yl)boronic acid (**F$_n$B** (*n* = 1–4)) (1.6 equiv) and (A-^{ta}Phos)$_2$PdCl$_2$ (0.1 equiv) were dissolved in dry tetrahydrofuran (20 mL). An aqueous solution of K_3PO_4 (1.44 M, 1.5 equiv per boronic acid functionality) was added and the reaction mixture was degassed and heated up to 70 °C. It was refluxed under nitrogen for several hours. The reaction mixture was quenched with water, diluted in dichloromethane and washed with brine and water. The combined organic fractions were dried over $MgSO_4$ and the solvent was evaporated. The crude product was purified by column

chromatography on silica gel eluting with hexane:dichloromethane (10:3). The product was dissolved in the minimum amount of dichloromethane and precipitated from methanol to yield a dark green powder.

3,7-Bis(9,9-dihexyl-9*H*-fluoren-2-yl)-10-(4-(9,9-dihexyl-9*H*-fluoren-2-yl)phenyl)-5,5-difluoro-5*H*-dipyrrolo[1,2-*c*:2',1'-*f*][1,3,2]diazaborinin-4-ium-5-uide (**Y-B1**)

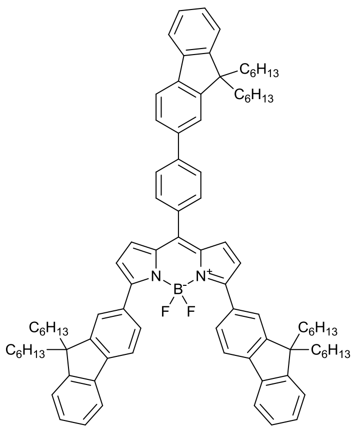

Using the general procedure B, the quantities used were: (**Y-B0Hal**) (150 mg, 0.361 mmol), 9,9'-dihexylfluorenyl-2-boronic acid (**F_1B**) (651 mg, 1.72 mmol), (A-^{ta}Phos)$_2$PdCl$_2$ (77 mg, 0.11 mmol), K_3PO_4 1.44 M (1.80 mL, 2.59 mmol). The reaction was refluxed under nitrogen for 36 hours. The product was obtained as a dark green powder (122 mg, 0.0964 mmol, 27%). ^{1}H NMR (CD_2Cl_2, *δ*, 400 MHz): 8.06 (2 H, dd, ^3J = 8.2 Hz, ^4J = 1.4 Hz), 7.90 (2 H, d, *J* = 8.4 Hz), 7.88-7.83 (3 H, m), 7.81-7.70 (9 H, m), 7.45-7.30 (9 H, m), 7.07 (2 H, d, ^3J = 4.4 Hz), 6.82 (2 H, d, ^3J = 4.0 Hz), 2.20-1.90 (12 H, m), 1.20-0.94 (36 H, m), 0.82-0.55 (30 H, m). ^{13}C NMR (CD_2Cl_2, *δ*, 100 MHz): 159.43, 152.17, 151.97, 151.56, 151.03, 143.91, 143.29, 143.01, 141.72, 141.02, 140.96, 139.24, 137.07, 133.76, 131.82, 131.71, 130.92, 129.00, 127.99, 127.78, 127.37, 127.31, 127.25, 126.45, 124.55, 123.48, 123.44, 122.03, 121.49, 120.59, 120.56, 120.28, 119.78, 55.73, 55.68, 40.80, 40.61, 31.97,

31.90, 30.13, 30.08, 24.30, 24.18, 23.00, 22.94, 14.18. (MALDI/TOF, m/z): [M^+] calcd. for $C_{90}H_{107}BF_2N_2$: 1265.6; found 1264.2.

5,5-Difluoro-3,7-bis(9,9,9',9'-tetrahexyl-9*H*,9'*H*-[2,2'-bifluoren]-7-yl)-10-(4-(9,9,9',9'-tetrahexyl-9*H*,9'*H*-[2,2'-bifluoren]-7-yl)phenyl)-5*H*-dipyrrolo[1,2-*c*:2',1'-*f*][1,3,2]diazaborinin-4-ium-5-uide (**Y-B2**)

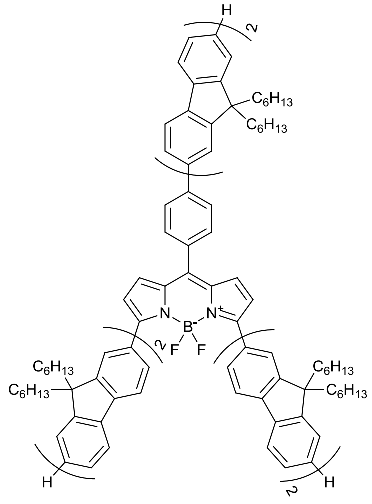

Using the general procedure B, the quantities used were: (**Y-B0Hal**) (100 mg, 0.240 mmol), (9,9,9',9'-tetrahexyl-2,2'-bifluoren-7-yl)boronic acid (**F$_2$B**) (815 mg, 1.15 mmol), (A-^{ta}Phos)$_2$PdCl$_2$ (51 mg, 0.072 mmol, K_3PO_4 1.44 M (1.20 mL, 1.73 mmol). The reaction was refluxed under nitrogen for 48 hours. The product was obtained as a dark green powder (82 mg, 0.036 mmol, 15%). ^{1}H NMR (CD_2Cl_2, δ, 400 MHz): 8.12 (2 H, d, ^3J = 8.0 Hz), 7.99-7.62 (32 H, m), 7.47-7.28 (9 H, m), 7.10 (2 H, d, ^3J = 4.4 Hz), 6.86 (2 H, d, ^3J = 4.4 Hz), 2.30-1.95 (24 H, m), 1.24-1.00 (72 H, m), 0.87-0.60 (60 H, m). ^{13}C NMR (CD_2Cl_2, δ, 100 MHz): 159.41, 152.77, 152.49, 152.36, 151.98, 151.94, 151.50, 151.35, 143.91, 143.22, 142.73, 141.55, 141.43, 141.31, 141.23, 140.89, 140.86, 140.83, 140.23, 140.18, 139.25, 137.13, 133.80, 131.86, 131.73, 130.94, 129.15, 127.47, 127.39, 127.22, 126.58, 126.44, 124.62, 123.42, 122.08, 121.99, 121.96, 121.53, 120.89, 120.66, 120.58, 120.27, 120.10,

119.88, 55.92, 55.87, 55.67, 55.65, 40.83, 40.75, 40.63, 31.96, 31.93, 31.90, 30.08, 24.37, 24.26, 23.01, 22.96, 14.22, 14.18. (MALDI/TOF, m/z): [M^+] calcd. for $C_{165}H_{203}BF_2N_2$: 2263.2; found 2262.1.

5,5-Difluoro-3,7-bis(9,9,9',9',9'',9''-hexahexyl-9*H*,9'*H*,9''*H*-[2,2':7',2''-terfluoren]-7-yl)-10-(4-(9,9,9',9',9'',9''-hexahexyl-9*H*,9'*H*,9''*H*-[2,2':7',2''-terfluoren]-7-yl)phenyl)-5*H*-dipyrrolo[1,2-*c*:2',1'-*f*][1,3,2]diazaborinin-4-ium-5-uide (**Y-B3**)

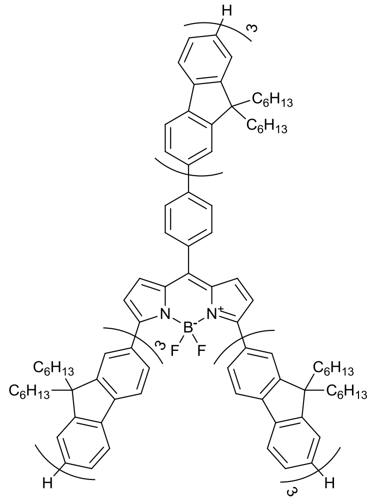

Using the general procedure B, the quantities used were: (**Y-B0Hal**) (150 mg, 0.361·mmol), (9,9,9',9',9'',9''-hexahexyl-9*H*,9'*H*,9''*H*,9'''*H*-2,2':7',2''-terfluoren-7-yl)boronic acid (**F_3B**) (1.80 g, 1.73·mmol), (A-^{ta}Phos)$_2$PdCl$_2$ (77 mg, 0.11 mmol), K_3PO_4 1.44 M (1.79 mL, 2.58 mmol). The reaction was refluxed under nitrogen for 48 hours. The product was obtained as a dark green powder (356 mg, 0.109 mmol, 30%). ^{1}H NMR (CD_2Cl_2, δ, 400 MHz): 8.12 (2 H, d, ^3J = 8.4 Hz), 7.98-7.64 (50 H, m), 7.45-7.27 (9 H, m), 7.11 (2 H, d, ^3J = 4.0 Hz), 6.87 (2 H, d, J = 4.4 Hz), 2.30-1.90 (36 H, m), 1.25-1.10 (108 H, m), 0.86-0.60 (90 H, m). ^{13}C NMR (CD_2Cl_2, δ, 100 MHz): 159.44, 152.82, 152.53, 152.40, 152.30, 151.97, 151.50, 151.39, 143.94, 142.75, 141.56, 141.27, 141.01, 140.93, 140.84, 140.61, 140.47, 140.24, 139.28, 137.17, 131.89, 131.77, 127.47, 127.24, 126.58, 126.42, 124.68, 123.44, 122.02, 121.92,

120.93, 120.41, 120.30, 120.10, 119.92, 55.90, 55.86, 55.68, 40.79, 31.99, 31.96, 31.93, 30.11, 24.35, 24.30, 23.04, 23.00, 14.21. (MALDI/TOF, m/z): [M^+] calcd. for $C_{240}H_{299}BF_2N_2$: 3260.8; found 3260.0.

5,5-Difluoro-3,7-bis(9,9,9',9',9'',9'',9''',9'''-octahexyl-9*H*,9'*H*,9''*H,*9'''*H*-[2,2':7',2'':7'',2'''-quaterfluoren]-7-yl)-10-(4-(9,9,9',9',9'',9'',9'''-octahexyl-9*H*,9'*H*,9''*H*,9'''*H*-[2,2':7',2'':7'',2'''-quaterfluoren]-7-yl)phenyl)-5*H*-dipyrrolo[1,2-*c*:2',1'-*f*][1,3,2]diazaborinin-4-ium-5-uide (**Y-B4**)

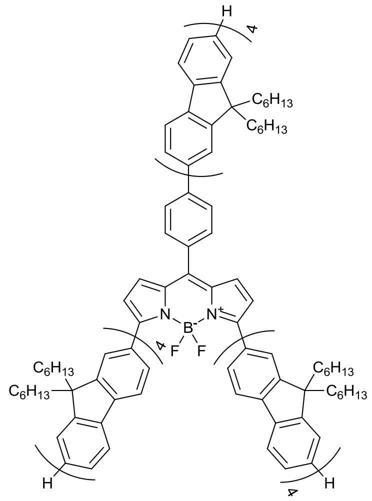

Using the general procedure B, the quantities used were: (**Y-B0Hal**) (85 mg, 0.204·mmol), (9,9,9',9',9'',9'',9''',9'''-octahexyl-9*H*,9'*H*,9''*H*,9'''*H*,9''''*H*-2,2':7',2'':7'',2'''-quaterfluoren-7-yl)boronic acid (**F_4B**) (1.34 g, 0.974 mmol), $(A\text{-}^{ta}Phos)_2PdCl_2$ (44 mg, 0.062 mmol), K_3PO_4 1.44 M (1.02 mL, 1.47 mmol). The reaction was refluxed under nitrogen for 24 hours. The product was obtained as a dark green powder (62 mg, 0.0146 mmol, 7.1%). ^{1}H NMR (CD_2Cl_2, δ, 400 MHz): 8.13 (2 H, d, ^3J = 8 Hz), 8.00-7.64 (68 H, m), 7.47-7.27 (9 H, m), 7.12 (2 H, d, ^3J = 4.0 Hz), 6.88 (2 H, d, ^3J = 4.4 Hz), 2.30-1.90 (48 H, m), 1.27-1.00 (144 H, m), 0.90-0.60 (120 H, m). ^{13}C NMR (CD_2Cl_2, δ, 100 MHz): 159.43, 152.82, 152.52, 152.40, 152.29, 151.97, 151.49,

151.39, 143.94, 142.75, 141.56, 141.26, 140.99, 140.93, 140.84, 140.61, 140.49, 140.28, 140.24, 139.28, 137.16, 131.89, 131.77, 127.47, 127.23, 126.53, 126.41, 124.66, 123.44, 121.97, 121.92, 121.56, 120.94, 120.62, 120.39, 120.29, 120.09, 55.89, 55.86, 55.66, 40.78, 31.98, 31.94, 30.10, 24.35, 24.29, 22.98, 14.21. (MALDI/TOF, m/z): [M^+] calcd. for $C_{315}H_{395}BF_2N_2$: 4258.3; found: 4257.7.

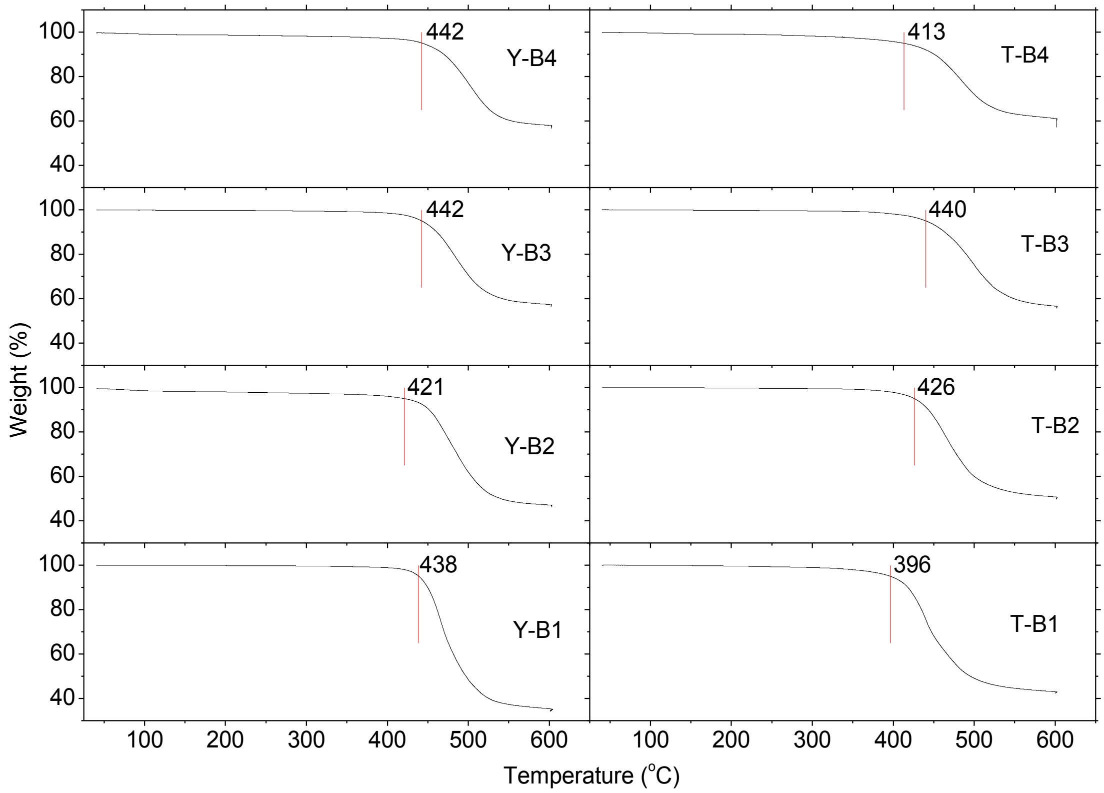

Figure S1: Thermogravimetric analysis of the BODYPI-oligofluorene compounds measured at 10 ºC/min; the temperature corresponding to 5% mass loss is shown.

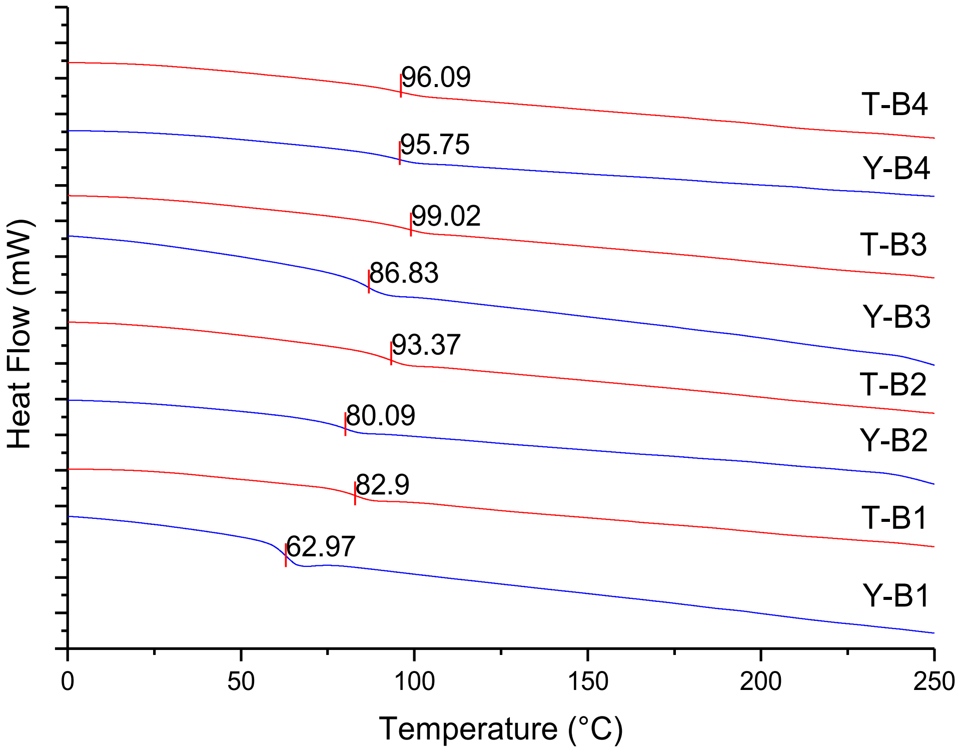

Figure S2: DSC analysis of BODIPY-oligofluorene compounds measured at 10 ^{o}C/min; the glass transition temperatures are indicated for each compound.

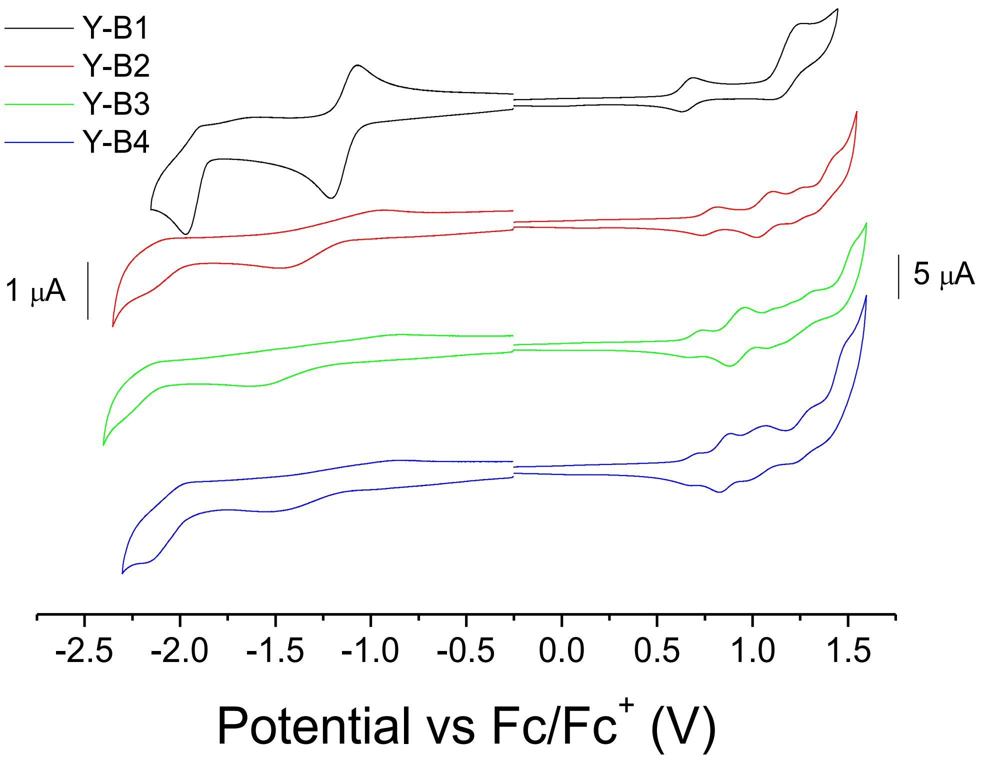

Figure S3: Cyclic voltammetry: oxidation waves (right) and reduction waves (left) of the **Y-Bn** (*n* = 1–4) series. Due to the broad irreversible nature of the 1st reduction waves for **Y-Bn** (*n* = 2–4) the current scale of the Y-axis for the reduction in these graphs is expanded.

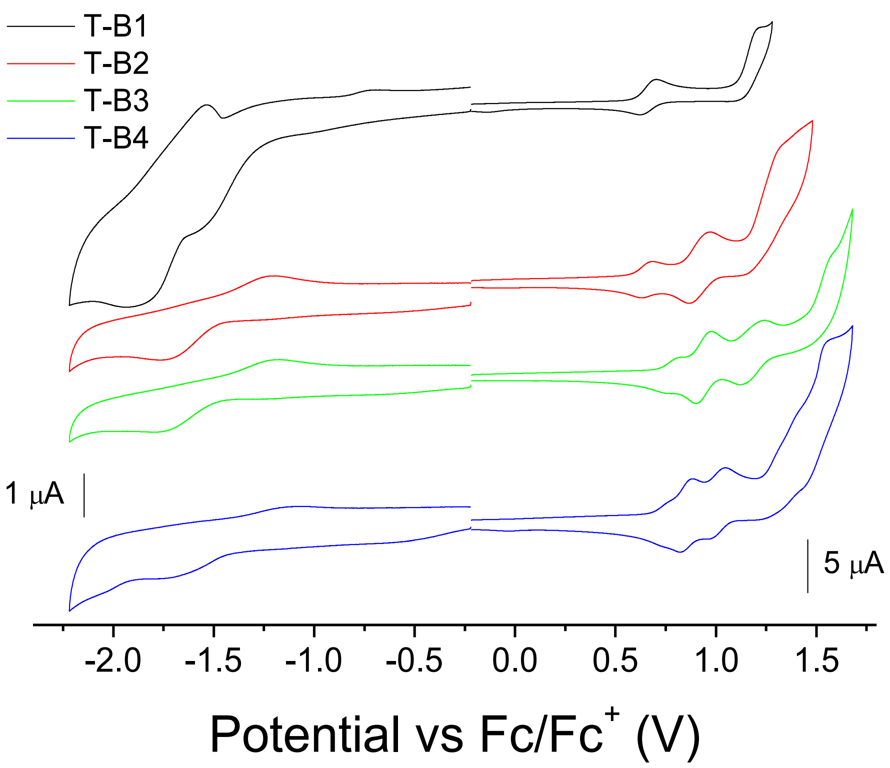

Figure S4: Cyclic voltammetry: oxidation waves (right) and reduction waves (left) of the **T-Bn** (*n* = 1–4) series. Due to the broad quasi-reversible/irreversible nature of the reduction waves the current scale of the Y-axis for the reduction processes is expanded with respect to the oxidation waves.

Table S1: Absorption and emission data for the **Y-Bn** (*n* = 1–4) and **T-Bn** (*n* = 1–4) series[a].

Compound	Absorption peaks positions, nm	Log(ε) t	Emission peak position, nm
**Y-B1**	317, 442, 599	4.83, 4.54, 4.80	646
**Y-B2**	346, 465, 612	5.20, 4.66, 4.81	661
**Y-B3**	360, 468, 614	5.38, 4.65, 4.78	664

**Y-B4**	369, 468, 614	5.54, 4.69, 4.81	663
**T-B1**	313, 399, 539	4.66, 4.00, 4.74	585
**T-B2**	341, 399 sh, 542	5.24, 4.23, 4.94	591
**T-B3**	358, 421 sh, 542	5.44, 4.25, 4.97	590
**T-B4**	367, 430 sh, 542	5.50, 4.11, 4.94	592

[a]The peaks of the most important absorption bands corresponding to the π – π*, S_0 – S_2 and S_0 – S_1 transitions are shown.

Table S2: Results of TDDFT calculations.

Compound	Calculated Absorption peaks, nm	$\Delta_{T\text{-}B1 - Y\text{-}B1\ CALC}$, nm ($\Delta_{T\text{-}B1 - Y\text{-}B1\ EXP}$, nm)	Transitions
**T-B1**	489		HOMO-3 -> LUMO (20%)<br>HOMO -> LUMO (80%)
	331		HOMO-10 -> LUMO (22%)<br>HOMO-7 -> LUMO (16%)<br>HOMO-2 -> LUMO (35%)<br>HOMO-1 -> LUMO (27%)
	290		HOMO-2 -> LUMO+1 (79%)<br>HOMO-1 -> LUMO+1 (21%)
	281		HOMO-6 -> LUMO (10%)<br>HOMO-4 -> LUMO (12%)<br>HOMO-3 -> LUMO (14%)<br>HOMO-3 -> LUMO+2 (17%)<br>HOMO-2 -> LUMO+3 (21%)<br>HOMO-1 -> LUMO+3 (6%)<br>HOMO -> LUMO+2 (19%)
**Y-B1**	540	0.24 (0.23)	HOMO-3 -> LUMO (20%)<br>HOMO -> LUMO (80%)
	367	0.36 (0.30)	HOMO-10 -> LUMO (15%)<br>HOMO-7 ->LUMO (14%)<br>HOMO-2 -> LUMO (20%)<br>HOMO-1 -> LUMO (43%)<br>HOMO-1 -> LUMO+1 (8%)
	288		HOMO-13 -> LUMO (85%)<br>HOMO-1 -> LUMO+1 (15%)

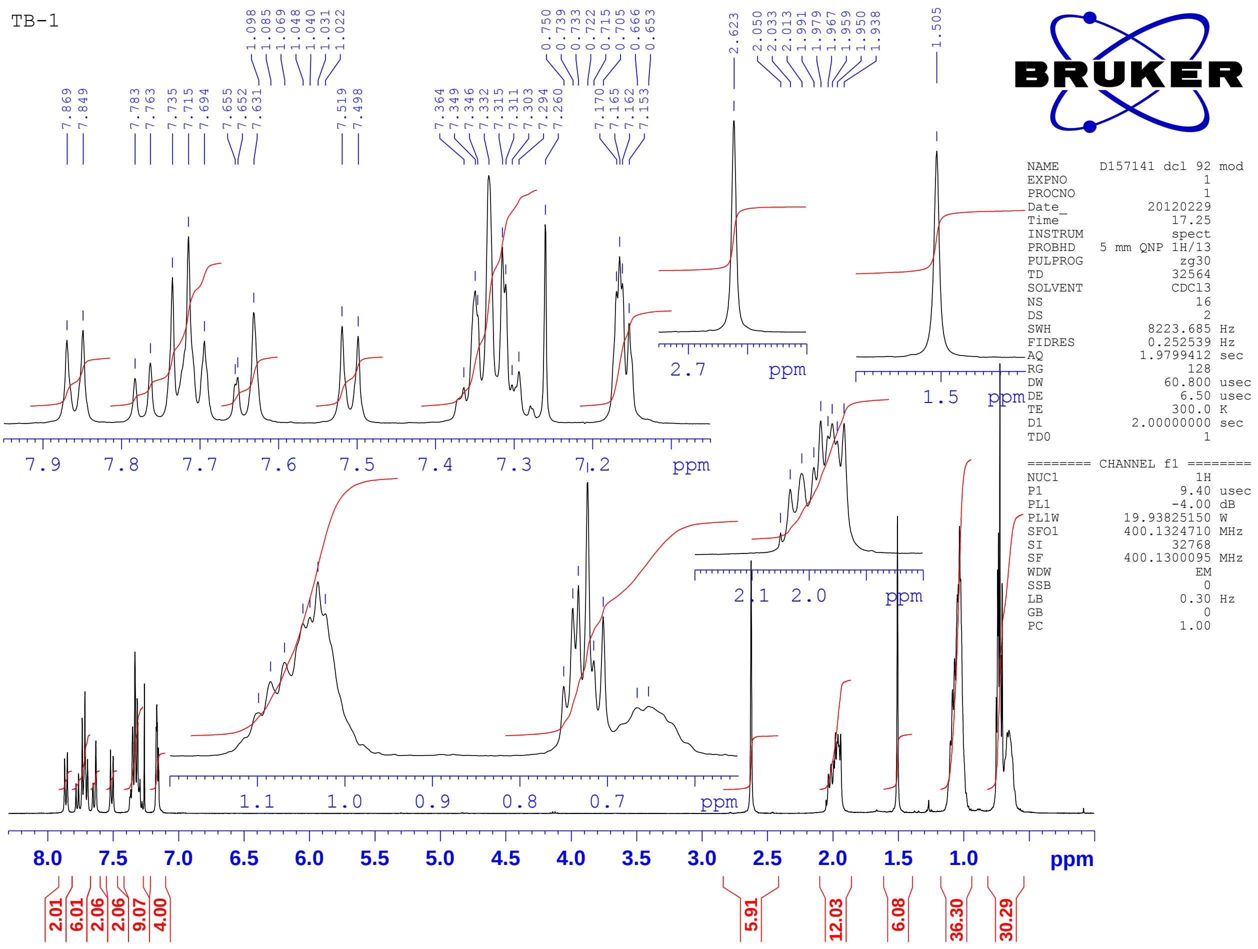

Figure S12: ^{1}H NMR of **T-B1** in $CDCl_3$.

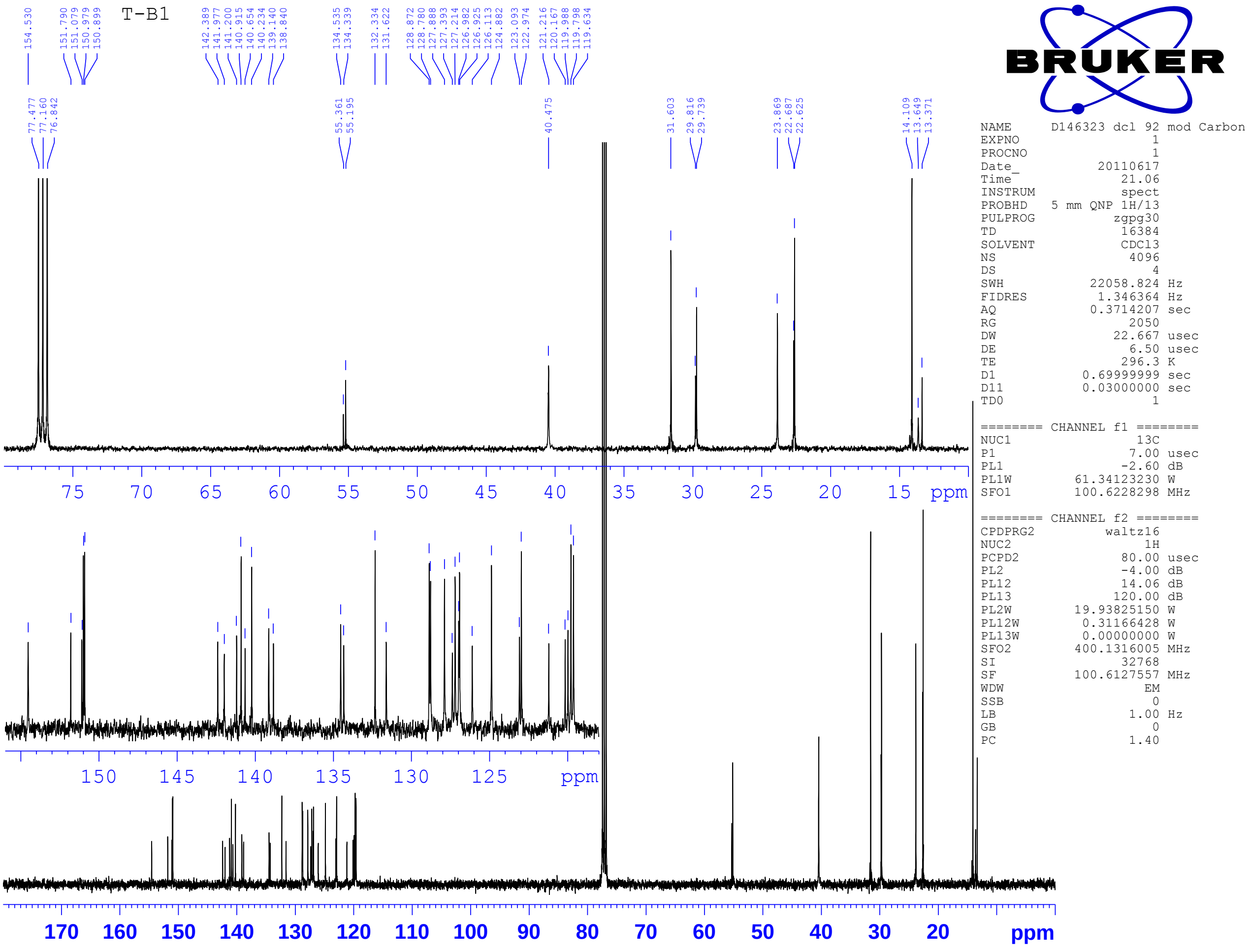

Figure S13: ^{13}C NMR of **T-B1** in $CDCl_3$

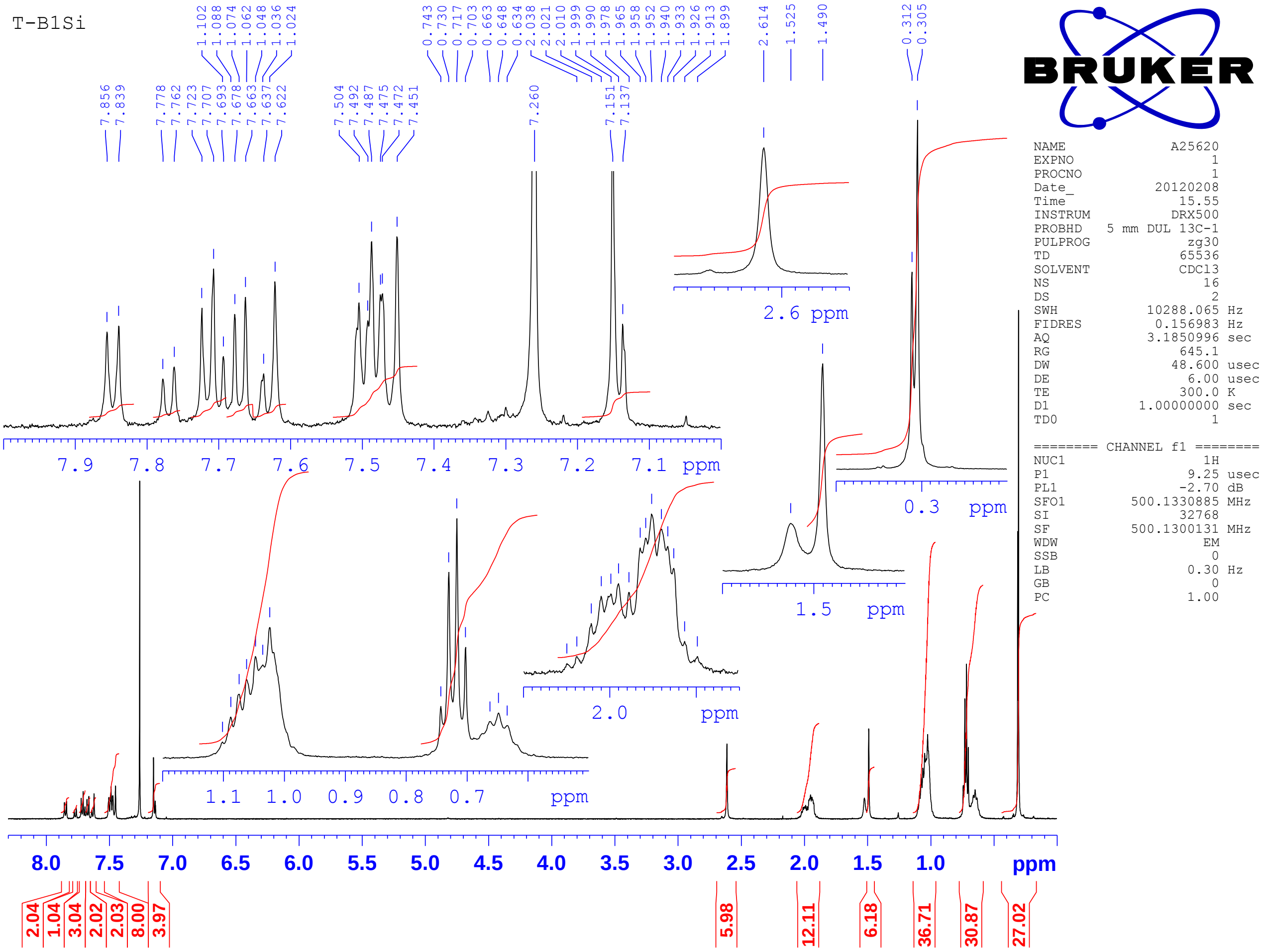

Figure S14: ^{1}H NMR of **T-B1Si** in $CDCl_3$.

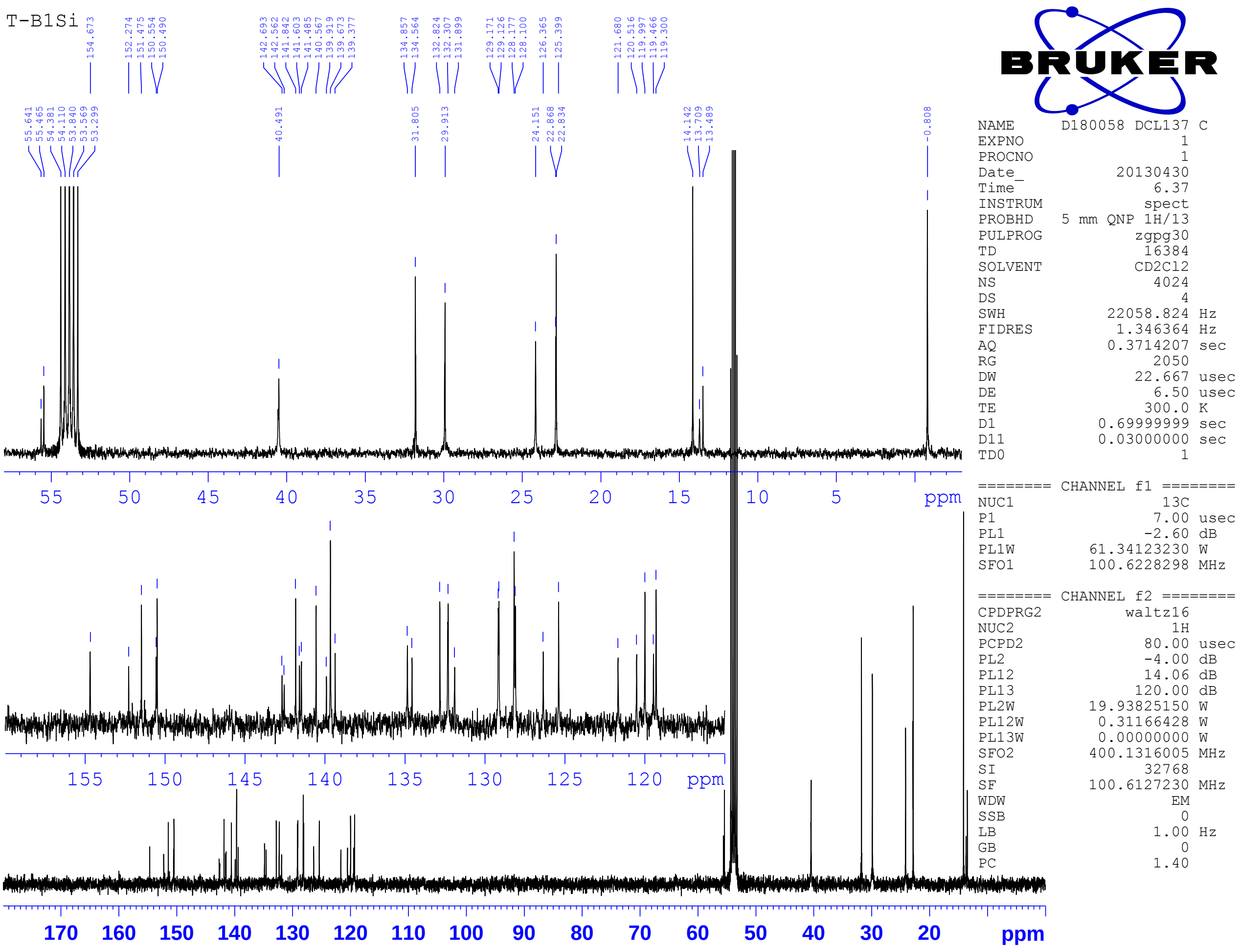

Figure S15: ^{13}C NMR of **T-B1Si** in CD_2Cl_2.

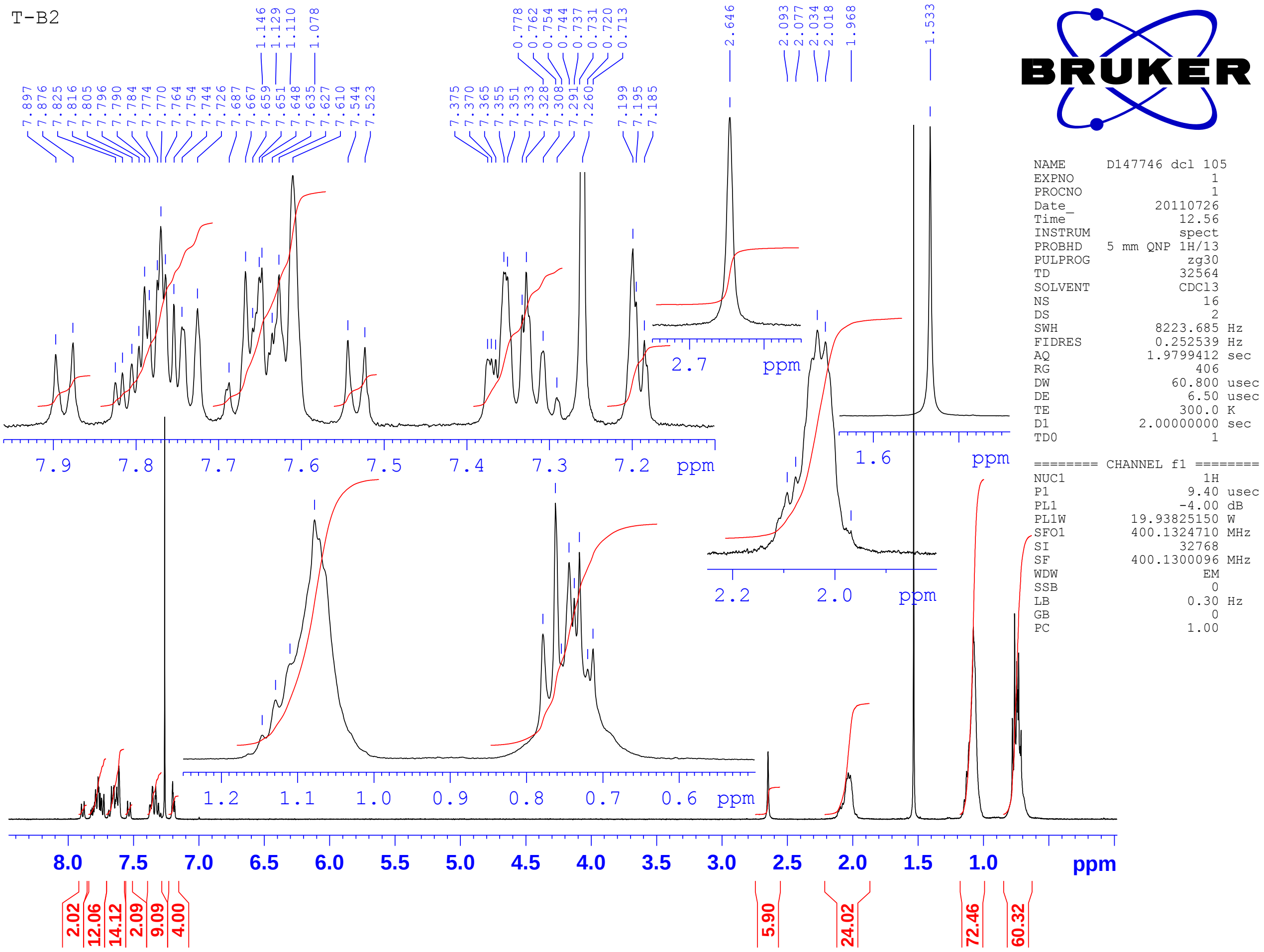

Figure S16: ^{1}H NMR of **T-B2** in $CDCl_3$.

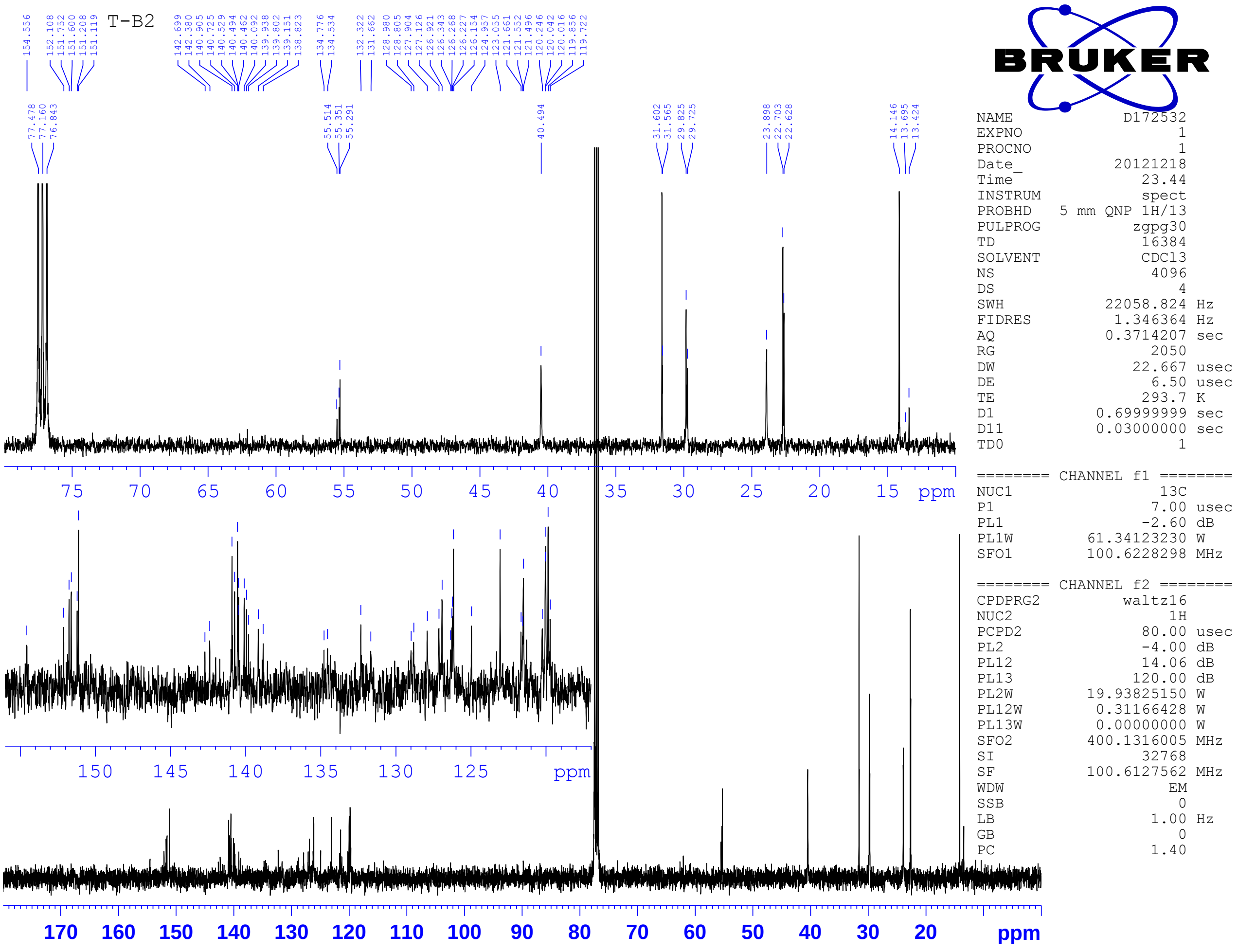

Figure S17: ^{13}C NMR of **T-B2** in $CDCl_3$.

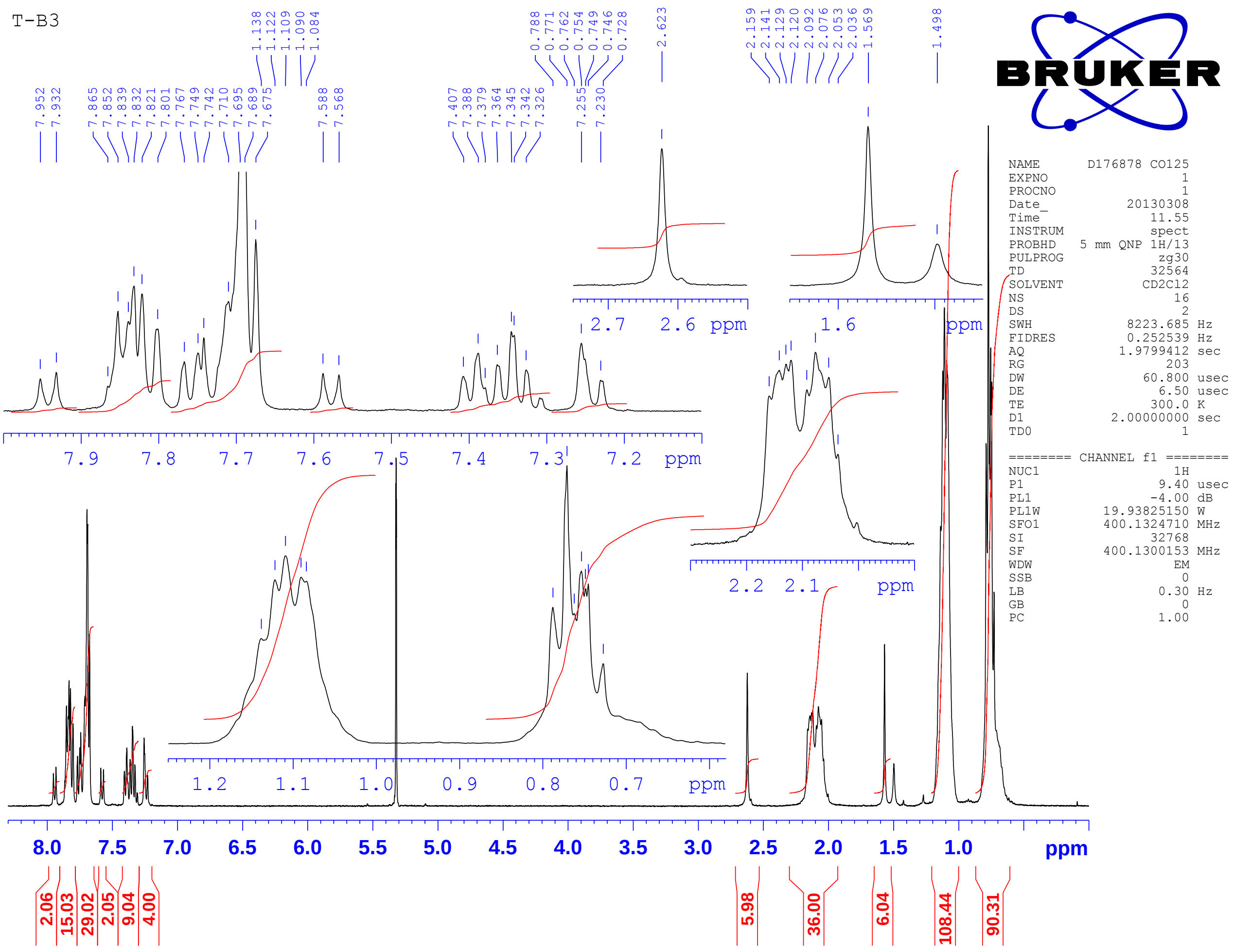

Figure S18: ^{1}H NMR of **T-B3** in CD_2Cl_2.

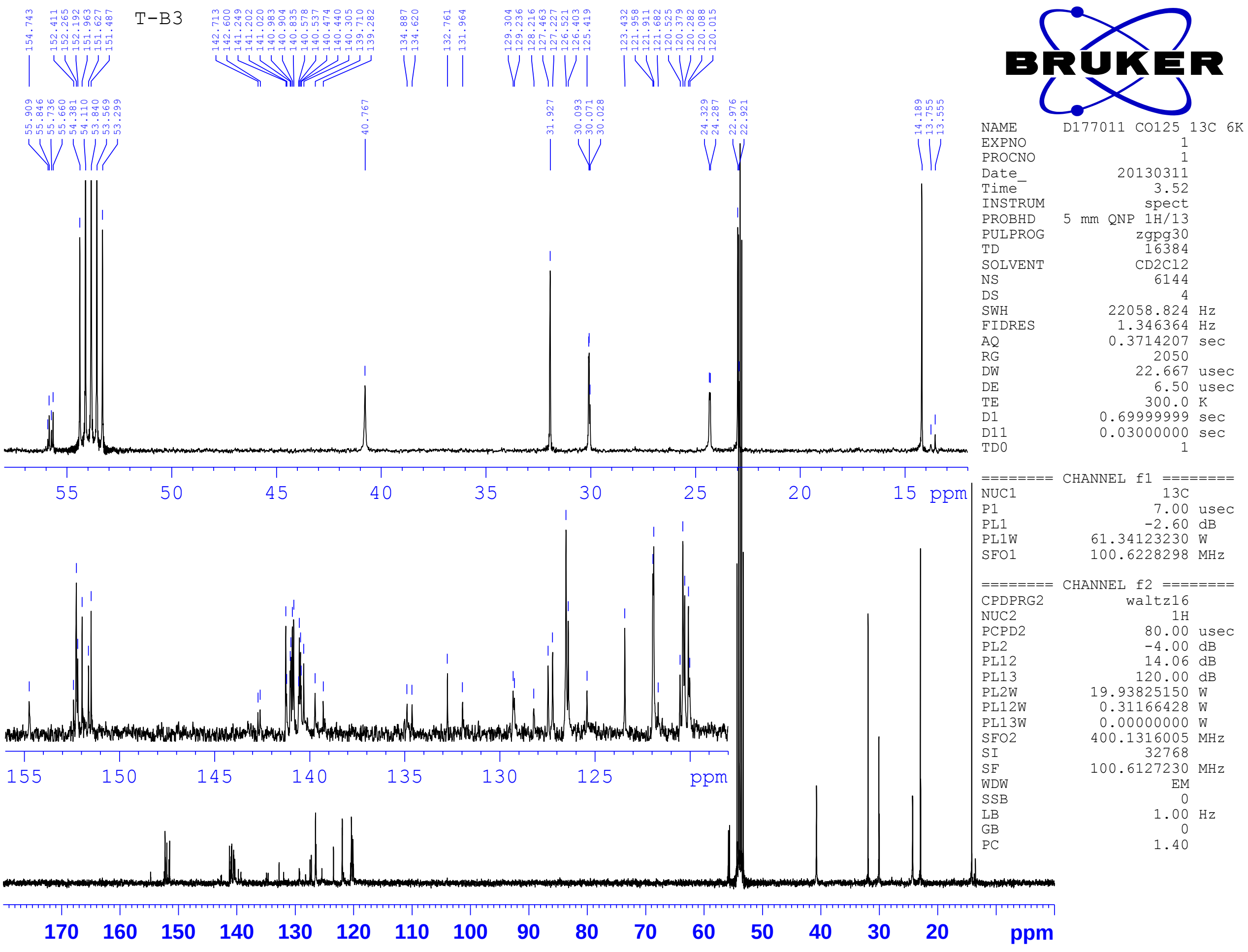

Figure S19: ^{13}C NMR of **T-B3** in CD_2Cl_2.

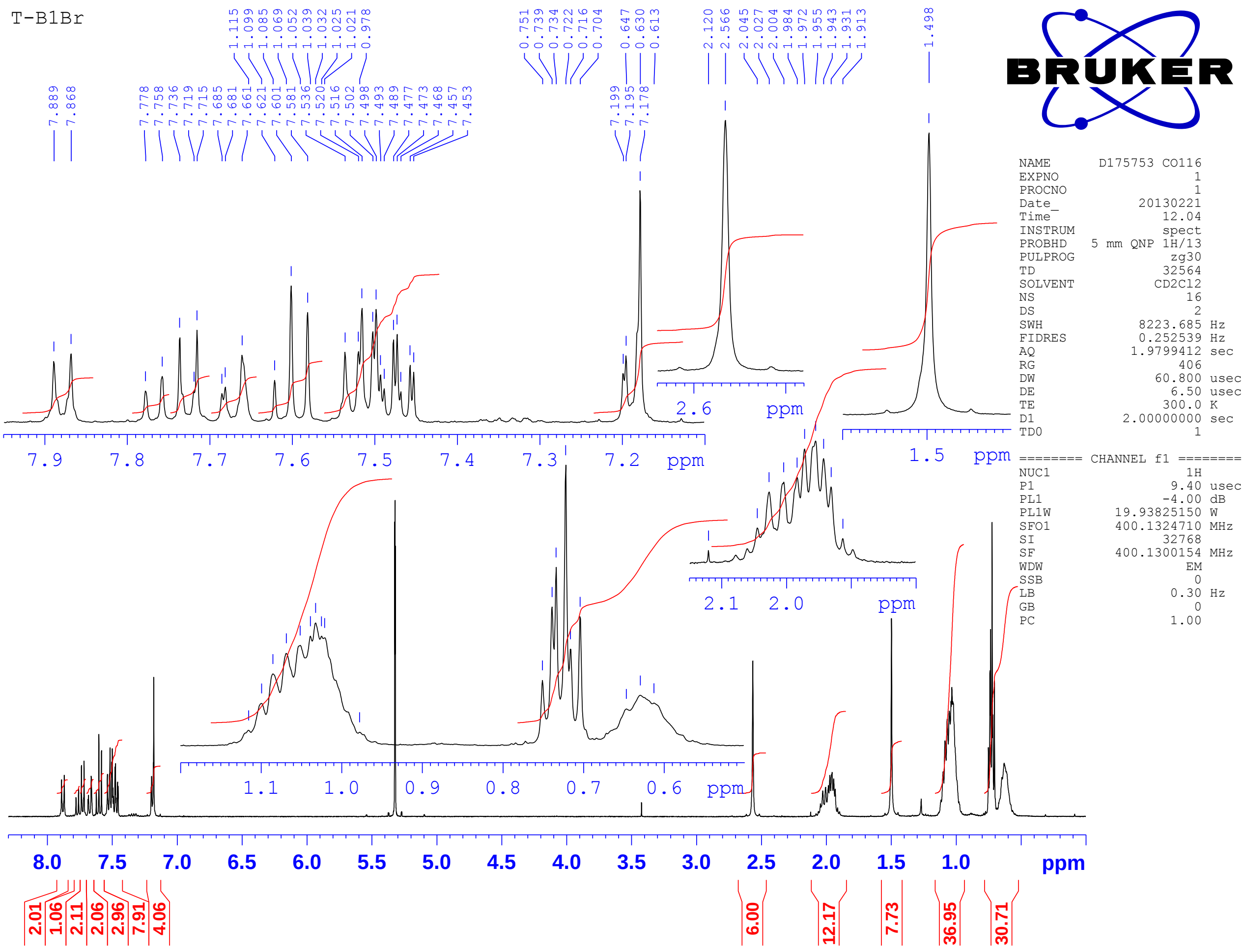

Figure S20: ^{1}H NMR of **T-B1Br** in CD_2Cl_2.

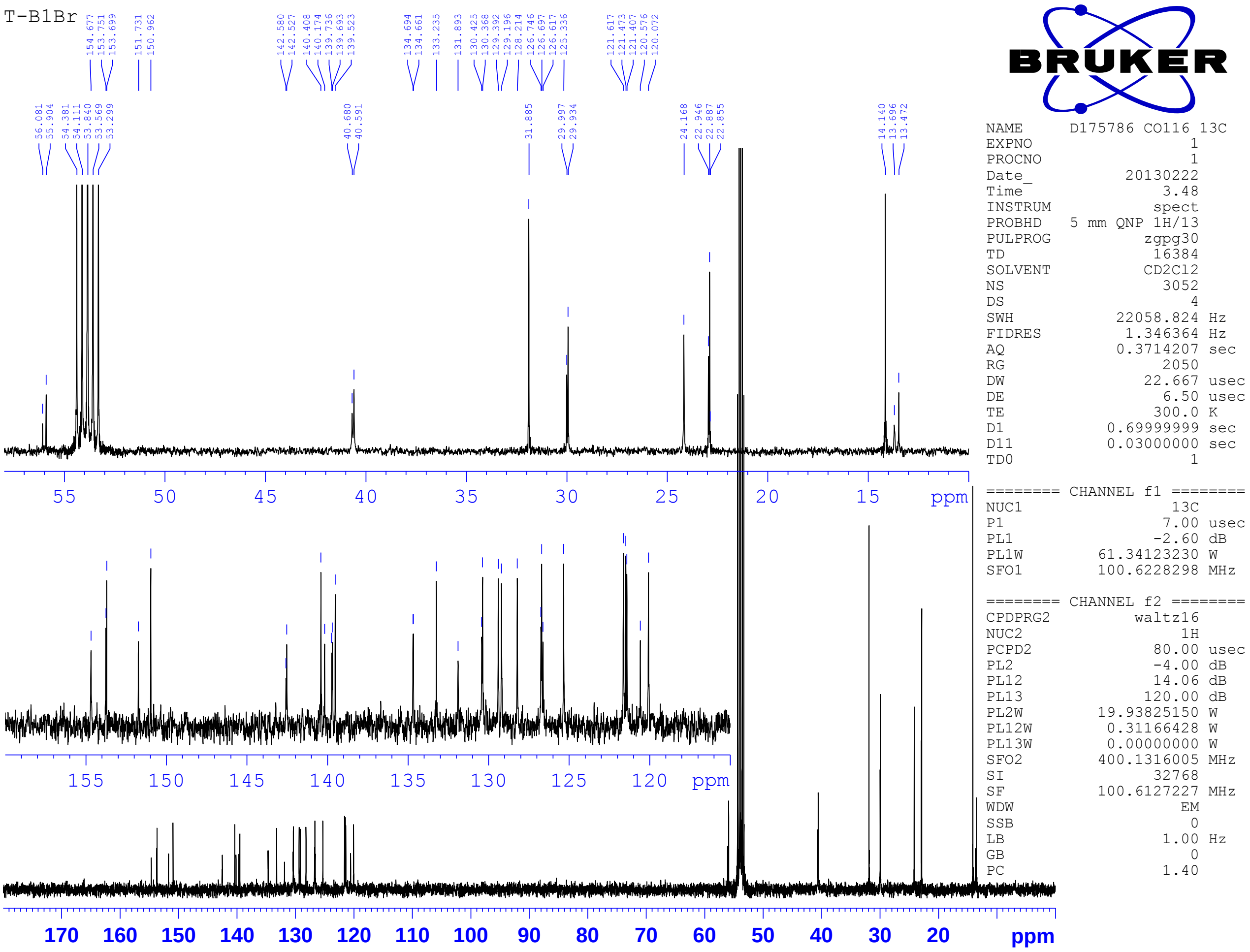

Figure S21: ^{13}C NMR of **T-B1Br** in CD_2Cl_2.

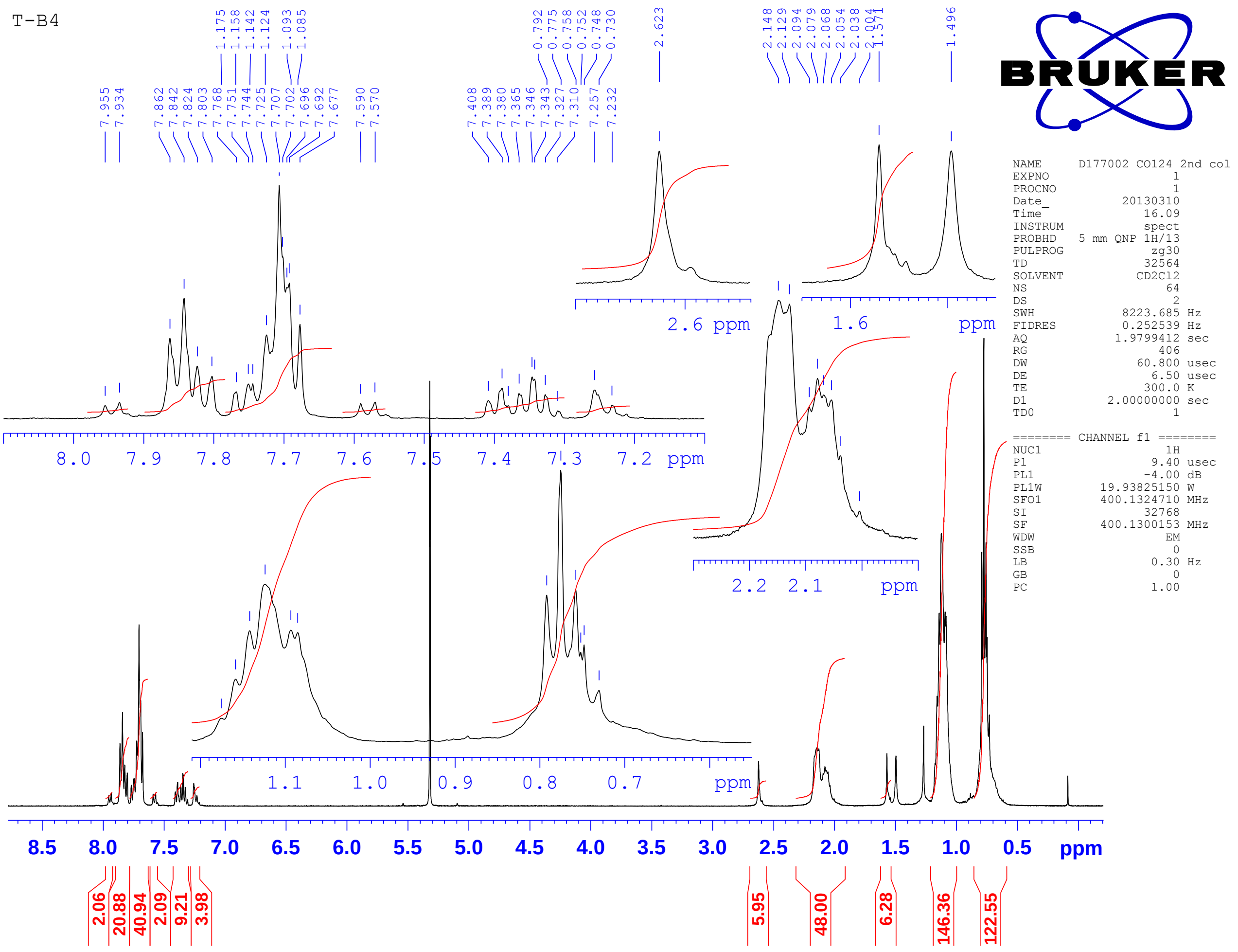

Figure S22: ^{1}H NMR of **T-B4** in CD_2Cl_2

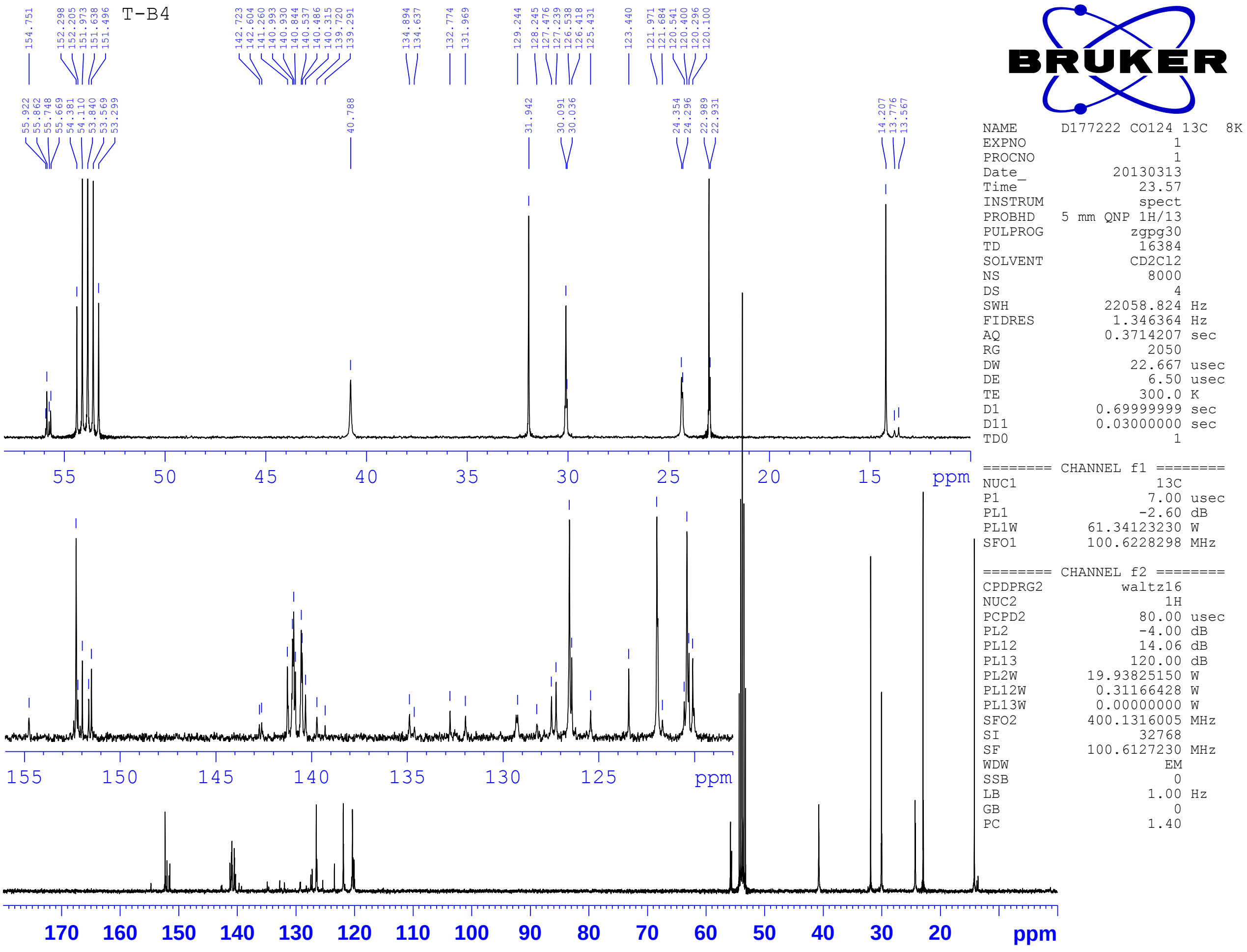

Figure S23: ^{13}C NMR of **T-B4** in CD_2Cl_2.

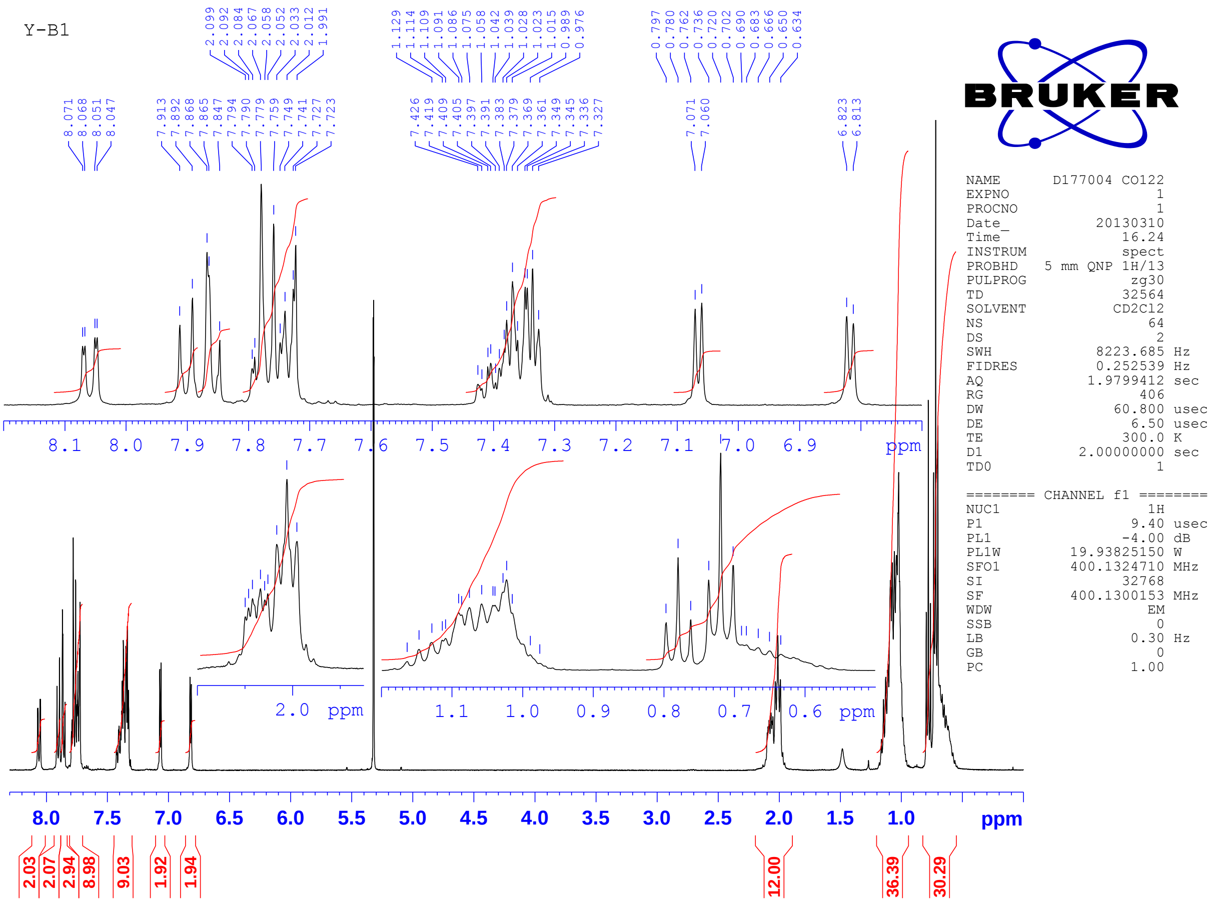

Figure S24: ^{1}H NMR of **Y-B1** in CD_2Cl_2.

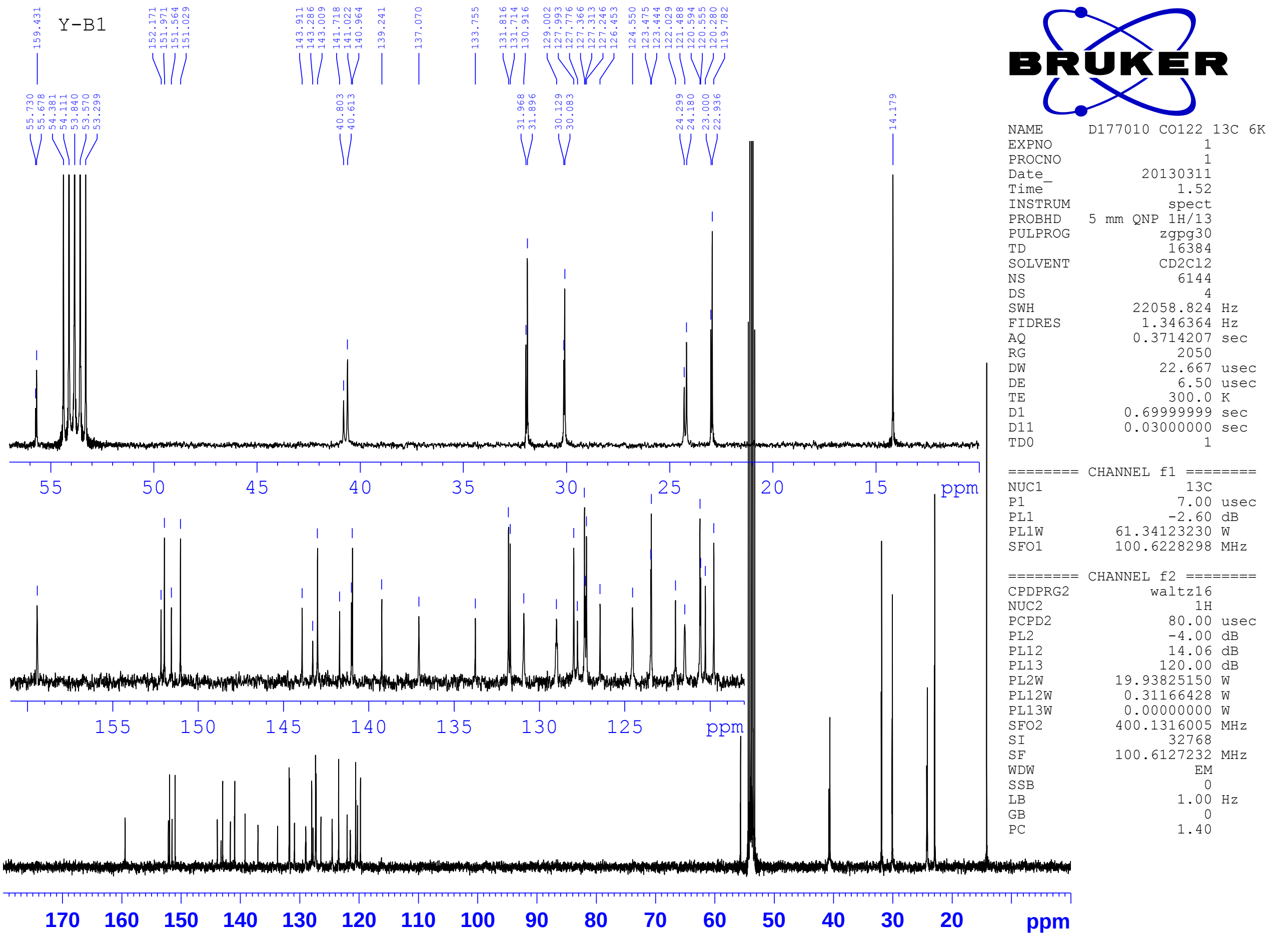

Figure S25: ^{13}C NMR of **Y-B1** in CD_2Cl_2.

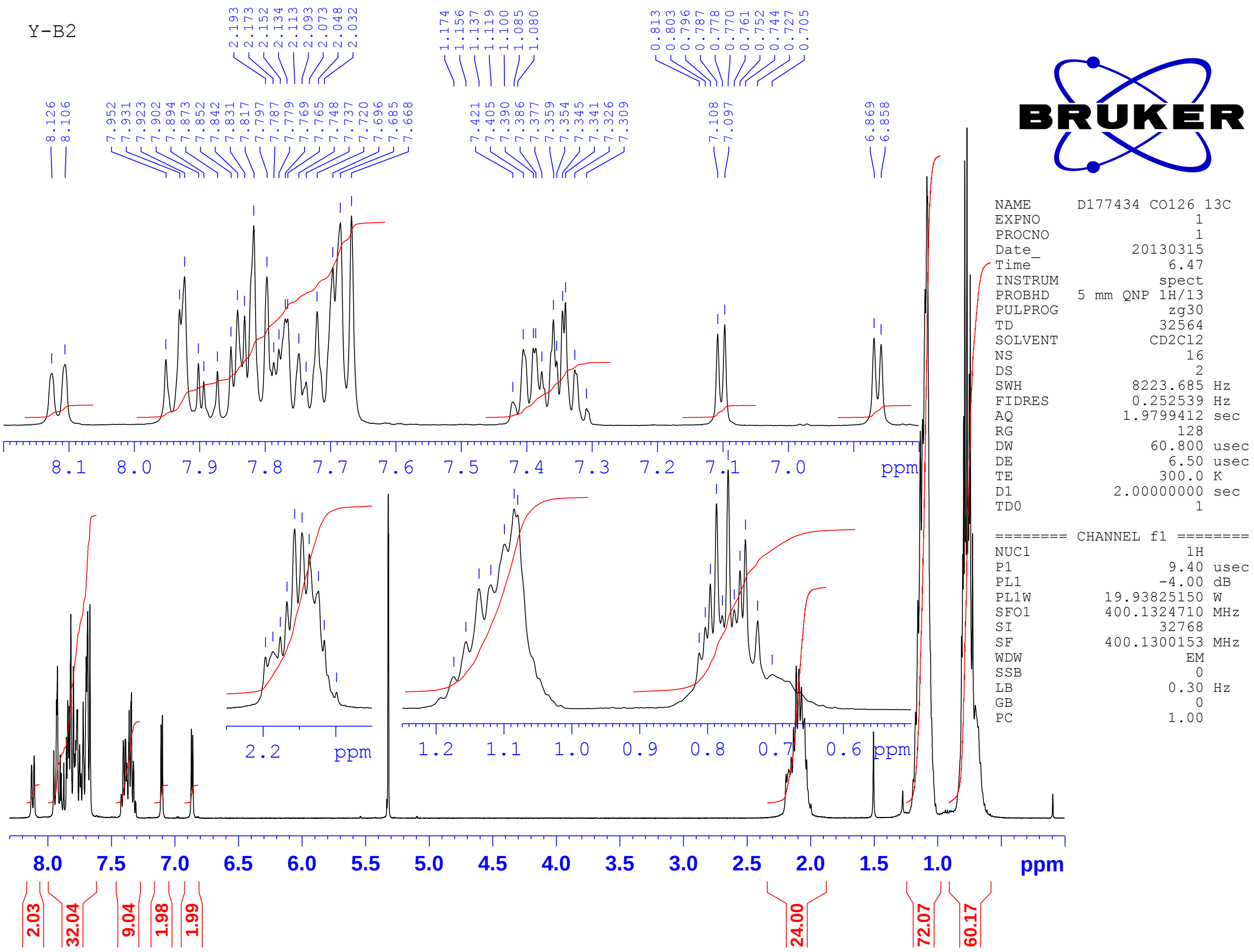

Figure S26: ^{1}H NMR of **Y-B2** in CD_2Cl_2.

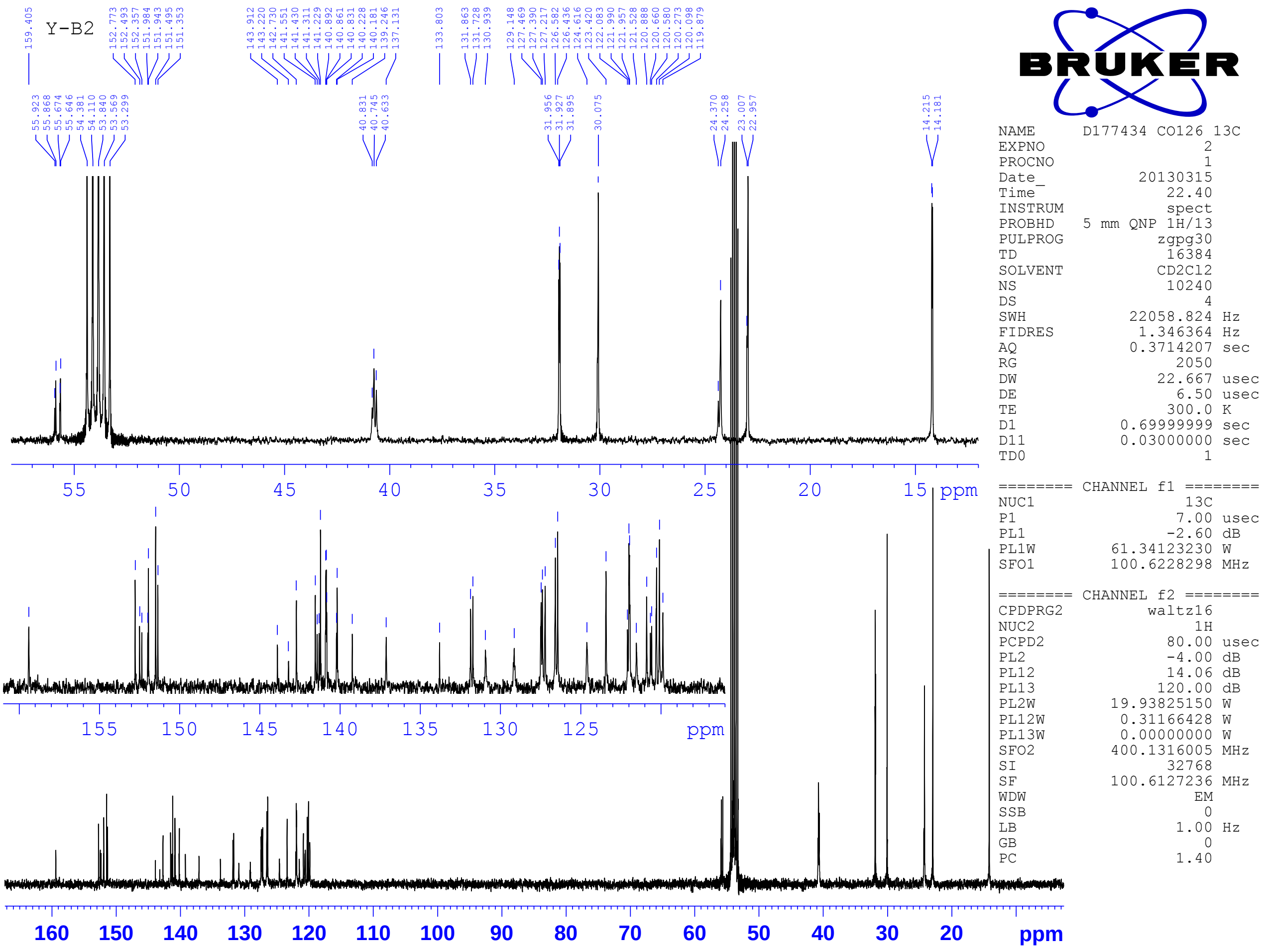

Figure S27: ^{13}C NMR of **Y-B2** in CD_2Cl_2.

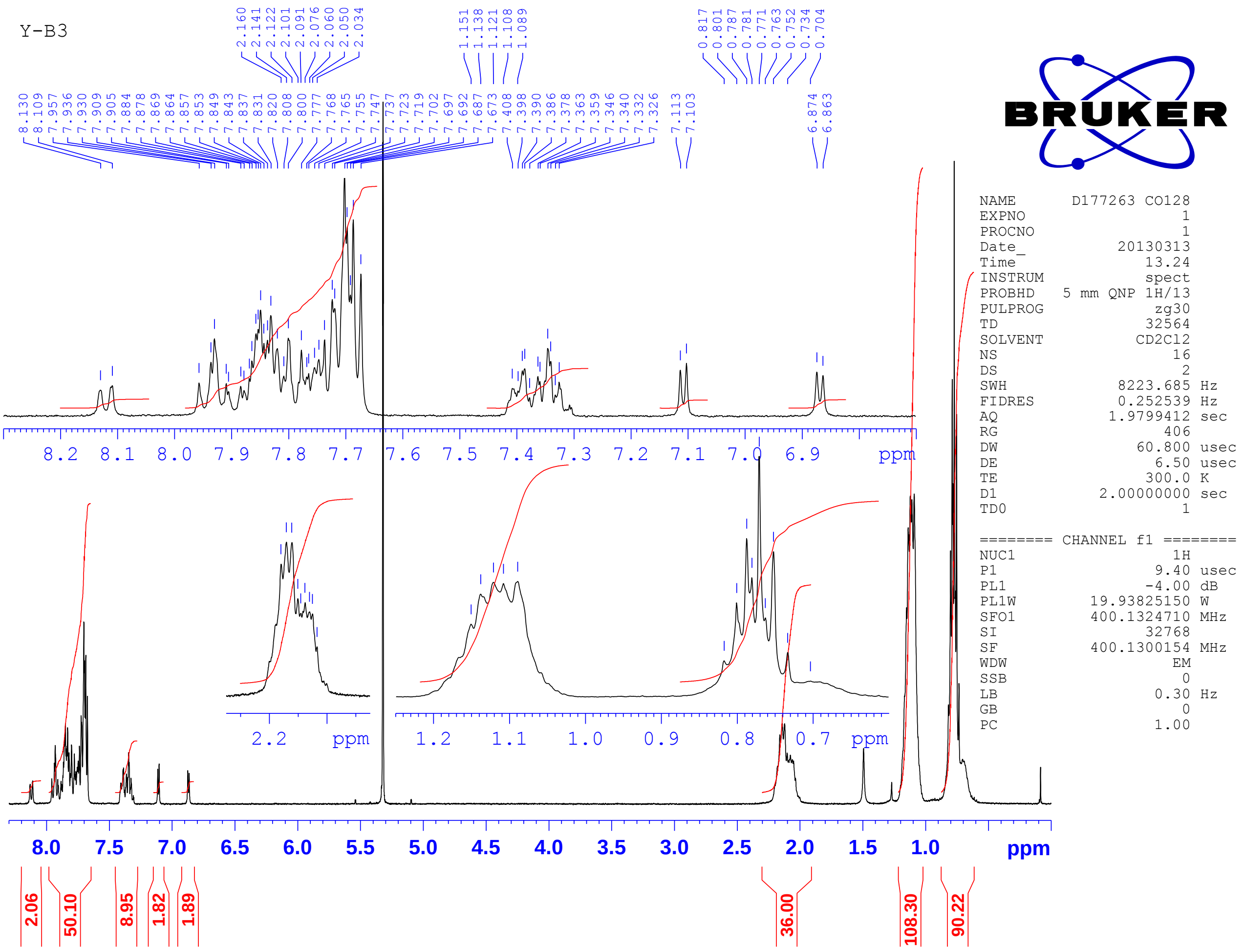

Figure S28: ^{1}H NMR of **Y-B3** in CD_2Cl_2.

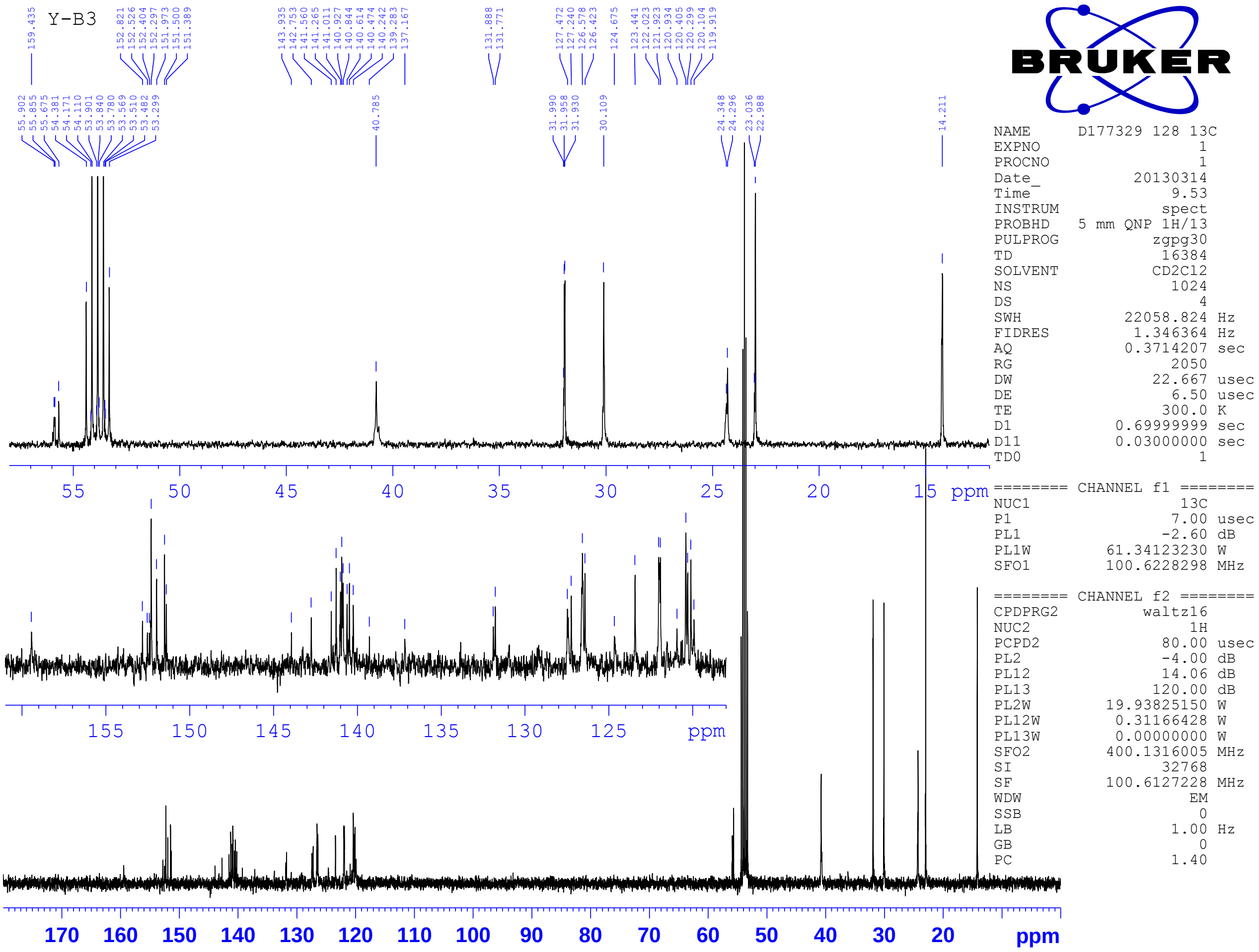

Figure S29: ^{13}C NMR of **Y-B3** in CD_2Cl_2.

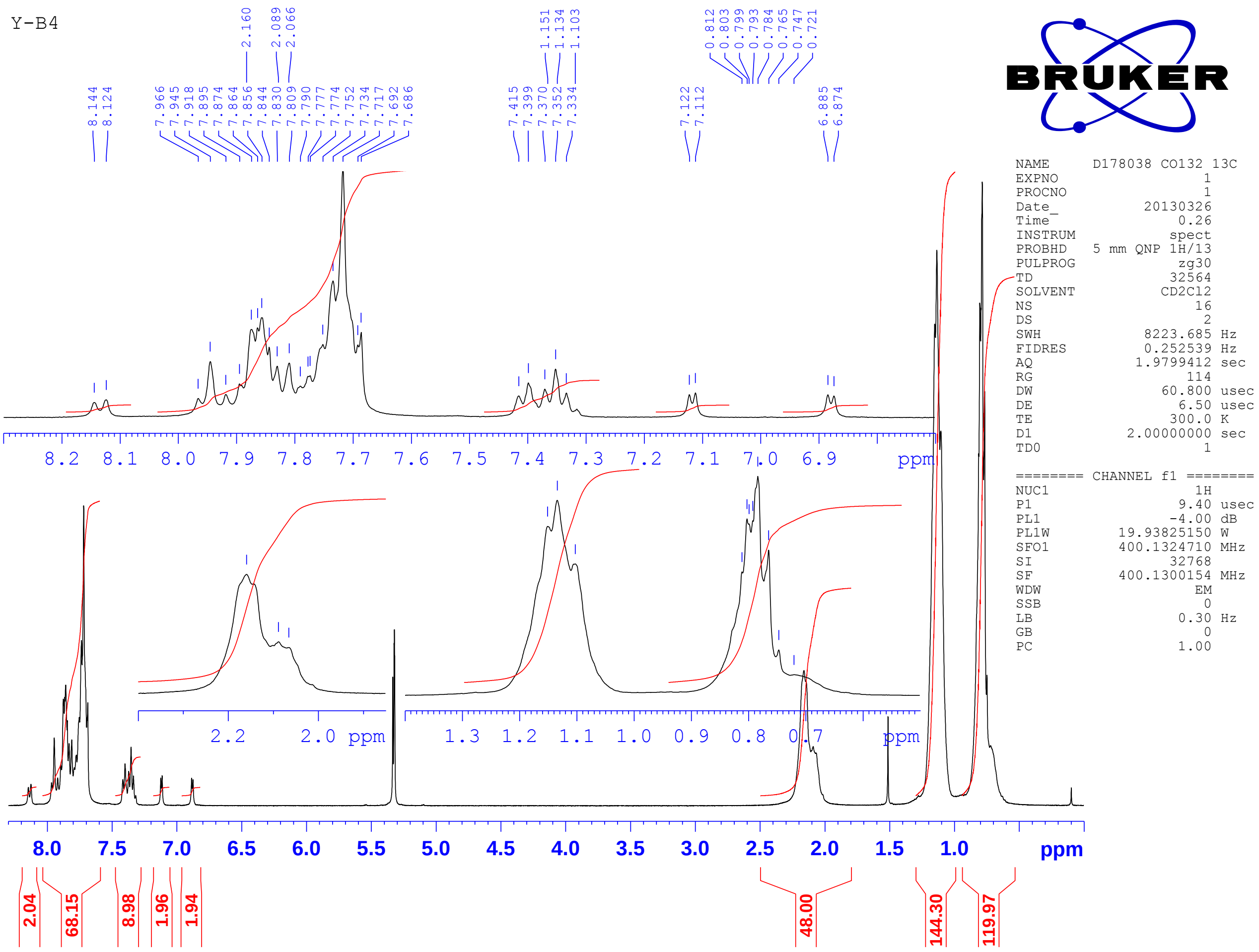

Figure S29: ^{1}H NMR of **Y-B4** in CD_2Cl_2.

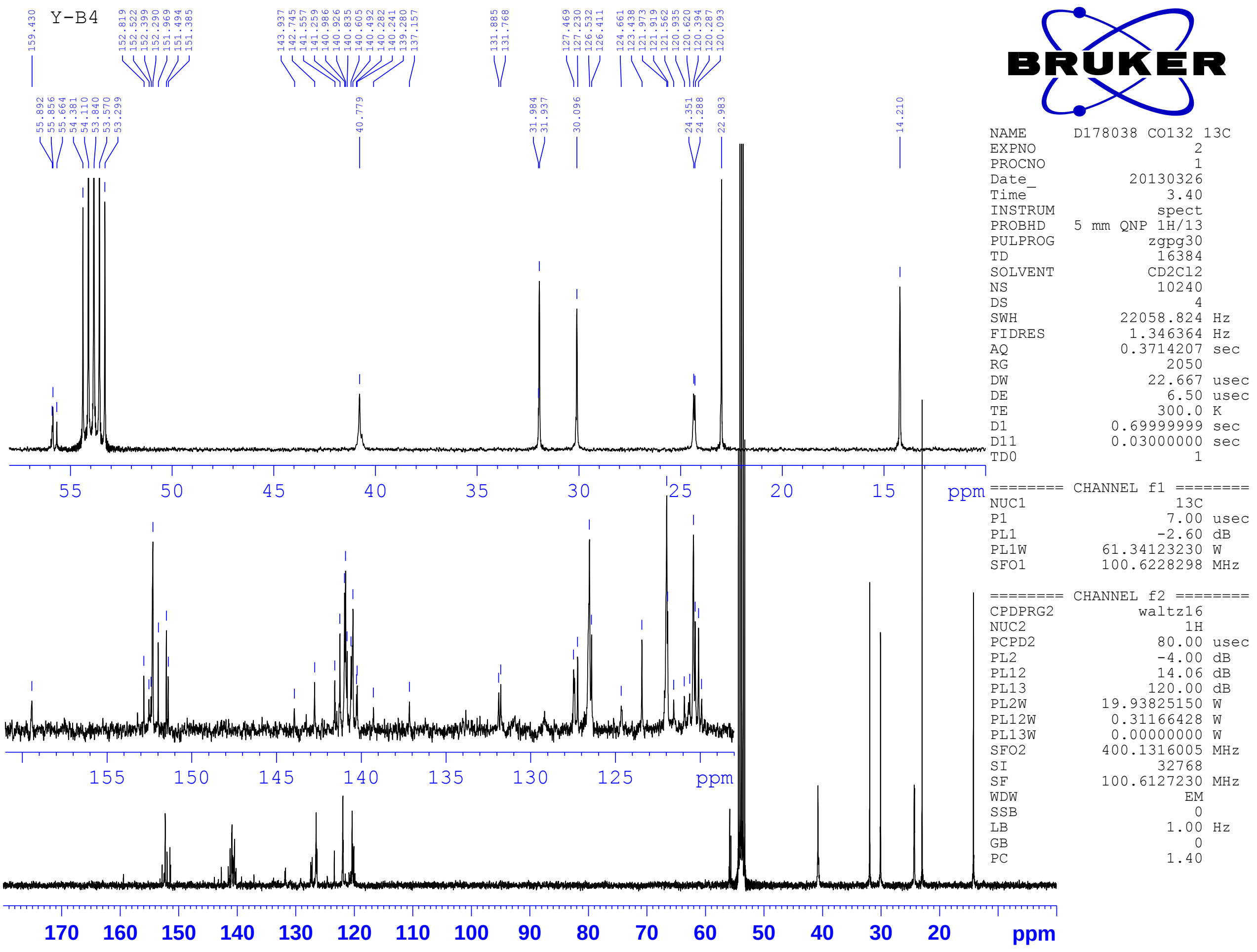

Figure S30: ^{13}C NMR of **Y-B4** in CD_2Cl_2.

References

[1] Zhang, X.; Xiao, Y.; Qian, X. *Org. Lett.* **2007,** *10,* 29–32. doi:10.1021/ol702381j

[2] Algi, M. P.; Tirkes, S.; Ertan, S.; Ergun, E. G. C.; Cihaner, A.; Algi, F. *Electrochim. Acta* **2013,** *109,* 766–774. doi:10.1016/j.electacta.2013.07.179
